# Gingerenone A Attenuates Ulcerative Colitis via Targeting IL‐17RA to Inhibit Inflammation and Restore Intestinal Barrier Function

**DOI:** 10.1002/advs.202400206

**Published:** 2024-04-19

**Authors:** Jian Liang, Weigang Dai, Chuanghui Liu, Yifan Wen, Chen Chen, Yifei Xu, Song Huang, Shaozhen Hou, Chun Li, Yongming Chen, Wei Wang, Hailin Tang

**Affiliations:** ^1^ School of Pharmaceutical Sciences State Key Laboratory of Traditional Chinese Medicine Syndrome Guangzhou University of Chinese Medicine Guangzhou 510006 China; ^2^ State Key Laboratory of Oncology in South China Guangdong Provincial Clinical Research Center for Cancer Sun Yat‐Sen University Cancer Center Guangzhou 510060 China; ^3^ Dongguan Institute of Guangzhou University of Chinese Medicine Dongguan 523808 China; ^4^ Center of Ganstric Cancer The First Affiliated Hospital Sun Yat‐Sen University Guangzhou 510062 China; ^5^ Shenzhen Traditional Chinese Medicine Hospital The Fourth Clinical Medical College of Guangzhou University of Chinese Medicine Shenzhen 518033 China

**Keywords:** gingerenone A, il‐17RA signaling, inflammation, intestinal barrier, ulcerative colitis

## Abstract

Ulcerative colitis (UC) is a complicated and recurrent intestinal disease. Currently available drugs for UC treatment are scarce, therefore, novel therapeutic drugs for the UC are urgently to be developed. Gingerenone A (GA) is a phenolic compound known for its anti‐inflammatory effect, but its effect on UC remains unknown. Here, it is shown that GA protects mice against UC, which is closely associated with inhibiting intestinal mucosal inflammation and enhancing intestinal barrier integrity in vivo and in vitro. Of note, RNA sequencing analysis demonstrates an evident correlation with IL‐17 signaling pathway after GA treatment, and this effect is further corroborated by Western blot. Mechanistically, GA directly interacts with IL‐17RA protein through pull‐down, surface plasmon resonance analysis and molecular dynamics simulation. Importantly, lentivirus‐mediated IL‐17RA/Act1 knock‐down or GA co‐treatment with brodalumab/ixekizumab significantly impairs the protective effects of GA against DSS‐induced inflammation and barrier dysfunction, suggesting a critical role of IL‐17RA signaling for GA‐mediated protection against UC. Overall, these results indicate that GA is an effective agent against UC mainly through the direct binding of IL‐17RA to inhibit inflammatory signaling activation.

## Graphical abstract

This investigation demonstrates that Gingerenone A protected against DSS‐induced ulcerative colitis via inhibiting IL‐17 signaling and downstream inflammatory signaling activation to alleviate inflammation and enhance intestinal barrier function.

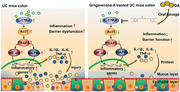

## 1 Introduction

Ulcerative colitis (UC) is a major type of inflammatory bowel diseases (IBDs), characterized by recurrent and diffuse intestinal mucosal inflammation, which extends continuously from the rectum to the proximal segment of the colon.^[^ ^1^ ^]^ Clinically, patients with UC have different degrees of systemic symptoms, including abdominal pain, recurrent diarrhea, rectal bleeding, body weight loss, arthritis and hepatic dysfunction, which affects patients’ life quality and economy.^[^ ^2^ ^]^ Importantly, UC has become the pivotal cause for the development of colorectal cancer (CRC).^[^ ^3^, ^4^, ^5^ ^]^ At present, Western countries have more UC patients, and the incidence of UC has also increased sharply in developing countries, making UC become a global health problem.^[^ ^6^ ^]^ Although the occurrence and development of UC are closely related to genetic, environmental and immune factors, its critical pathogenesis remains unclear.^[^ ^7^ ^]^ Currently, some drugs including aminosalicylic acid, sulfasalazine and immune‐suppressants have been clinically used to treat UC, but these medications can lead to drug tolerance and adverse reactions.^[^ ^8^, ^9^ ^]^ Therefore, it is urgent to search and develop more effective new drugs for the treatment of colitis.

Intestinal mucosal inflammation is considered to be an important player in the occurrence and development of UC, and it has become a key target for developing innovative anti‐colitis drugs.^[^ ^10^ ^]^ Recently, multitudinous studies have focused on the critical effect of IL‐17 signaling on immune‐inflammation related diseases.^[^ ^11^, ^12^ ^]^ The IL‐17 receptor (IL‐17R) family consists of five subunits, from IL‐17 receptor A (IL‐17RA) to receptor E (IL‐17RE). IL‐17A binds to the complex of IL‐17RA/IL‐17RC, and targeting IL‐17RA activates the pro‐inflammatory pathways to induce inflammatory response.^[^ ^13^ ^]^ Members of the IL‐17R family are defined by a conserved region in the cytoplasmic tail known as “SEF/IL‐17R” (SEFIR). The only other protein known to have SEFIR is the multifunctional adapter Act1, which has an effect on almost all known IL‐17 signaling events.^[^ ^14^ ^]^ Act1 contains a tumor necrosis factor receptor binding factor (Traf) motif that recruits different TRAFs to initiate separate downstream pathways. Upon activation, tumor necrosis factor receptor‐associated factor 6 (TRAF6) induces the mobilization of nuclear factor‐κB (NF‐κB) and mitogen‐activated protein kinase (MAPK) pathways (ERK, p38 and JNK), as well as up‐regulates numerous pro‐inflammatory cytokines (IL‐1β, IL‐6, and TNF‐α) and chemokines (CXCL1, CXCL2, CCL2, CCL7, COX2, and CCL20) to accelerate inflammatory response.^[^ ^12^, ^15^ ^]^ Actually, IL‐17 signaling is markedly activated in the colon of patients with IBDs or colitis mice.^[^ ^16^ ^]^ The requisite effect of IL‐17 signaling during the pathogenesis in dextran sulfate sodium (DSS)‐induced mouse UC model has been explored, which has evidences that the absence of IL‐17R/Act1/TRAF6 suppresses inflammation and remodels intestinal mucosal barrier in DSS‐induced colitis mice.^[^ ^12^, ^17^ ^]^ Therefore, searching for candidate drugs that targeting the inhibition of IL‐17 signaling can serve as a potential anti‐inflammatory strategy for UC treatment.

Natural products serve as a fundamental source for chemical diversity,^[^ ^18^ ^]^ which driving our exploration valuable active small molecules targeting IL‐17 signaling. Gingerenone A (GA), a phenolic compound, which is isolated from *Zingiber officinale*.^[^ ^19^ ^]^ Previous investigations have confirmed that GA possesses multiple pharmacological activities including anti‐hyperglycemic, anti‐obesity, hepatoprotection, anti‐tumor and anti‐inflammatory effects.^[^ ^19^, ^20^, ^21^ ^]^ Though there have been no relevant reports on the treatment of UC with GA, the efficacy of ginger in treating UC has been widely confirmed in clinic and animals models. Based on these, we hypothesized that GA might have therapeutic effects on UC. In this study, we investigated for the first time the effect of GA on alleviating DSS‐induced UC in mice and further explored its underlying mechanisms. We found that GA had a significant protective effect against DSS‐induced mucosal inflammation and barrier damage. Moreover, we confirmed that IL‐17RA as a pivotal molecular target of GA, which is able to improve intestinal mucosal inflammation and restore intestinal barrier homeostasis by inhibiting IL‐17RA signaling to ameliorate UC.

## 2 Results

### 2.1 GA Ameliorated DSS‐Induced Colitis Mouse Model and Inhibited Colitis‐Associated Colorectal Cancer

**Figure 1:**
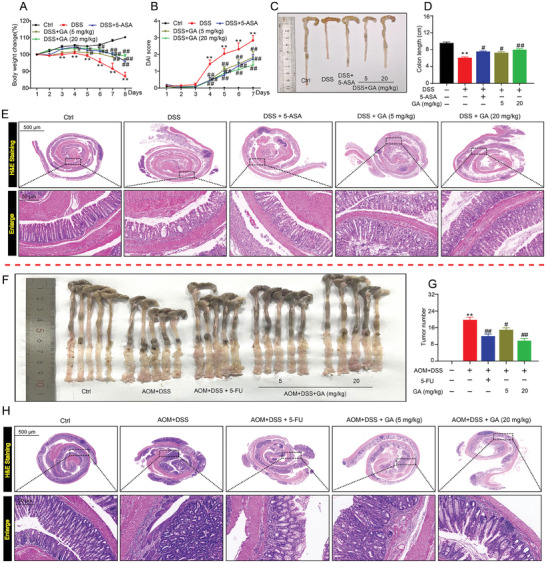
GA alleviates DSS‐induced mice colitis and inhibits colonic tumorigenesis. A) The change of body weight in each group for 7 days (*n* = 8). B) The DAI score in each group for 7 days (*n* = 10). C,D) Representative colon images and colon length (*n* = 8). E) Representative H&E images for colon tissues (*n* = 8). F) Representative pictures of colons with tumours (*n* = 5). G) Tumor number (*n* = 5). H) Representative H&E images of AOM/DSS model colonic tissues (*n* = 5). All data are expressed as mean ± S.E.M. ^*^P < 0.05 and ^**^P < 0.01 compared with the control group (Ctrl), *^#^P* < 0.05 and ^##^ *P* < 0.01 compared with the DSS group or AOM/DSS group.

To investigate the effect of GA on alleviating ulcerative colitis, C57BL/6 mice were challenged with 3% DSS for 7 days to induce an acute colitis model, which has analogous clinical symptoms (including body weight loss, diarrhea and bloody stool) in patients with UC.^[^ ^22^ ^]^ Meanwhile, colitis mice were orally administrated with GA (5 and 20 mg kg^−1^) or 5‐ASA (100 mg kg^−1^) for 7 days (Figure S1B, Supporting Information). The colitis mice experienced significant body weight loss on day 3 (**Figure** **1**A), with serious diarrhea and rectal bleeding beginning on day 4 (Figure 1B; Figure S1D,E, Supporting Information) until the end of the experiment. Compared with the DSS group mice, GA‐administrated colitis mice could significantly suppress body weight loss and reduce diarrhea, bloody stools and disease activity index (DAI) scores in a dose‐dependent manner (Figure 1A,B; Figure S1C–E, Supporting Information).

Colon shortening is often an important visual manifestation to reflect intestinal injury.^[^ ^23^ ^]^ Mice exposed to DSS could significantly reduce colon length. Whereas, GA‐treated colitis mice markedly protected against DSS‐induced colon shortening (Figure 1C,D). Consistently, the histopathological analysis of colon tissues indicated that colitis mice revealed serious pathological changes, including significant disappearance of goblet and epithelial cells, extensive inflammatory cell infiltration and extensively damaged crypt structures (Figure 1E; Figure S1F, Supporting Information). Conversely, GA treatment in DSS‐induced colitis mice significantly alleviated the pathological injury of colon tissue and reduced histopathological score, in a dose‐dependent manner (Figure 1E; Figure S1F, Supporting Information).

Since colitis is considered to be a high risk factor for colitis‐associated colorectal cancer (CAC), improving colitis plays an important role in the prevention and treatment of CAC.^[^ ^24^, ^25^, ^26^ ^]^ As expected, the average number of tumors was lower in the 5‐Fu or GA treatment groups as compared to the azoxymethane (AOM) + DSS group (Figure 1F,G). Moreover, we found that the numbers of large tumors (diameter > 4 mm) and middle tumors (2<diameter≤4) in AOM + DSS group were 28.28% and 36.35%, respectively, and 5‐Fu (2<diameter, 55.00%) or GA (2<diameter, 49.33% and 57.14%, respectively) treatment in CAC mice, the sizes of most tumor were lower than 2 mm (Figure S1H, Supporting Information), indicating that GA plays an important role in inhibiting CAC tumorigenesis. In addition, CAC mice treated with GA could significantly inhibit colonic atrophy (Figure S1I, Supporting Information), and histopathological analysis found that the dysplasia, inflammatory cell infiltration and intestinal epithelial destruction were markedly reduced in the GA‐treated colitis mice (Figure 1H; Figure S1J, Supporting Information). Overall, these results indicated that GA mitigated DSS‐induced colitis and inhibited colitis‐associated colorectal cancer.

### 2.2 GA Inhibited Mucosal Inflammatory Response and Maintained the Barrier Function in UC Mice

**Figure 2:**
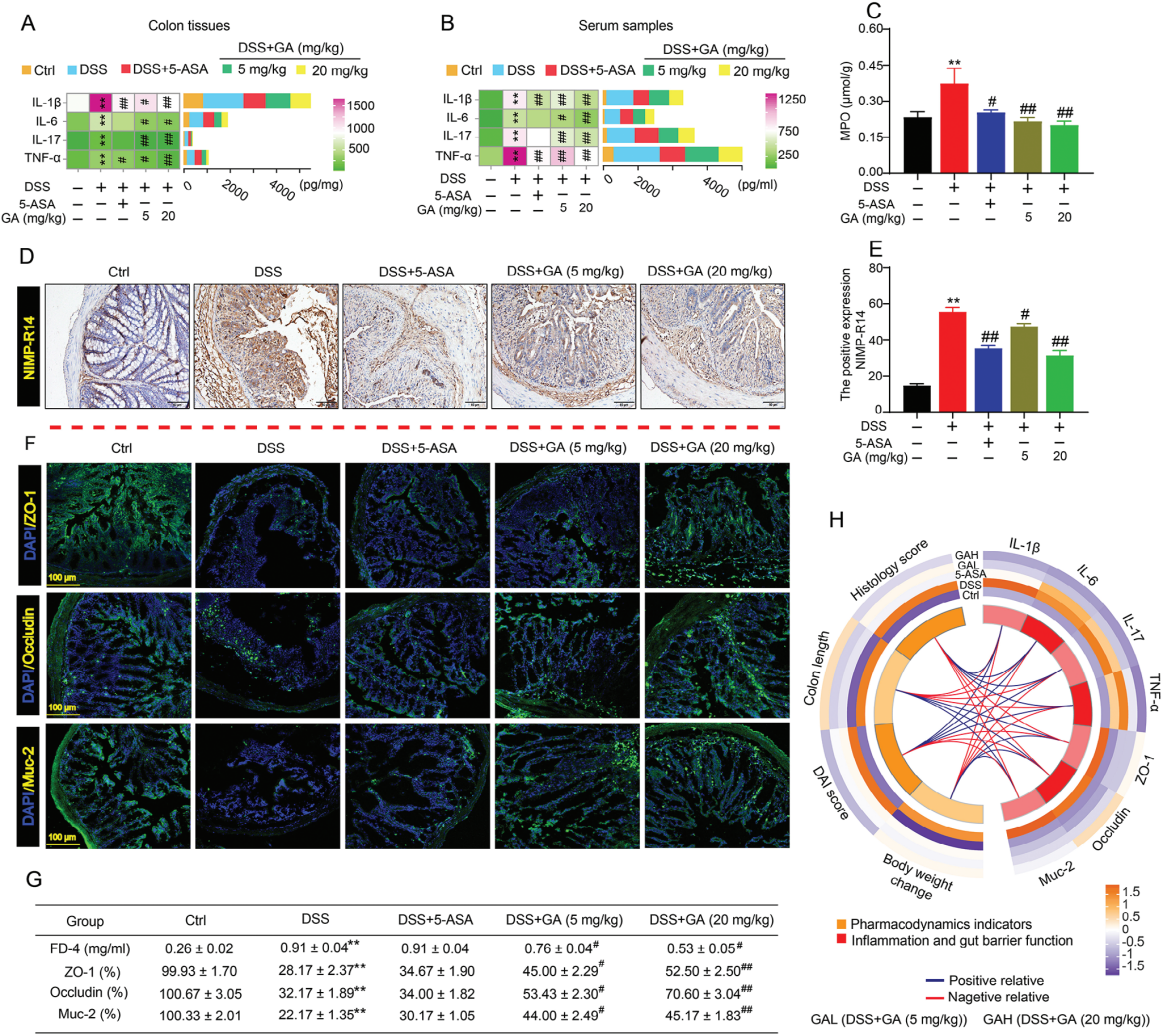
GA suppressed inflammation and protected against intestinal barrier dysfunction to attenuate colitis in mice. A) Inflammatory cytokines (IL‐1β, IL‐6, IL‐17, and TNF‐α) levels in colonic tissues (*n* = 6). B) Inflammatory cytokines (IL‐1β, IL‐6, IL‐17, and TNF‐α) levels in serum (*n* = 6). C) MPO activity in colonic tissues (*n* = 6). D,E) NIMP‐R14 positive expression level, and representative IHC pictures of NIMP‐R14 protein (*n* = 6). F) Representative immunofluorescence images of ZO‐1, Occludin, and Muc‐2 in colonic tissues (*n* = 3). G) Serum FD4 level (*n* = 6) and the fluorescence intensities of ZO‐1, Occludin, and Muc‐2 (*n* = 3). H) Pearson correlation heatmap circos among pro‐inflammation cytokines levels, intestinal barrier function proteins, and pharmacodynamic evaluation index in different groups (*n* = 3). All data are expressed as mean ± S.E.M. ^*^P < 0.05 and ^**^P < 0.01 compared with the control group (Ctrl), *^#^P* < 0.05 and ^##^ *P* < 0.01 compared with the DSS group.

As extensive mucosal inflammatory infiltration and barrier damage are the main pathological features of UC, including DSS‐induced animal models and UC patients.^[^ ^27^, ^28^ ^]^ We investigated whether GA alleviated intestinal mucosal inflammation and remodeled the intestinal barrier in DSS‐induced UC mice. As expected, we found that GA treatment in DSS‐induced UC mice significantly mitigated mucosal inflammatory response as evidenced by significantly decreasing the levels of IL‐1β, IL‐6, TNF‐α, IL‐17, and MPO in colonic tissues (**Figure** **2**A,C). Consistently, we also observed that GA could inhibit these pro‐inflammatory cytokines expressions in serum (Figure 2B). Besides, the result of IHC also indicated that a significant positive NIMP‐R14 expression was observed in the colon tissues of UC mice (Figure 2D,E). Importantly, GA treated in UC mice could dramatically blunted the expression of NIMP‐R14 (Figure 2D,E). These observations showed that GA could alleviate DSS‐induced colonic damage via suppressing intestinal mucosal inflammation.

As an important physical and chemical defense against heterogeneous antigens, the intestinal barrier is essential to maintain mucosal homeostasis.^[^ ^28^ ^]^ To explore the effect of GA on alleviating mucosal barrier function in DSS‐induced UC mice, oral administration of FITC‐dextran (FD‐4) was performed in mice to analyze the intestinal permeability. We found that DSS‐induced UC mice caused obvious intestinal mucosal damage, which confirmed by increasing the serum level of FD‐4 (Figure 2G), and this phenomenon was markedly reversed after treatment with GA (Figure 2G).

The epithelial cells and tight junctions are the major components in the intestinal barrier, which block paracellular pathways to exert its barrier function.^[^ ^29^, ^30^ ^]^ Considering that tissue damage is comparable to genotypic change, we concluded that the expression changes of intestinal tight junction proteins (such as ZO‐1, Occludin) may be the main cause for the increased permeability. Immunofluorescence analysis showed that mice exposed to DSS obviously reduced the expressions of ZO‐1 and Occludin proteins (Figure 2F,G). Importantly, GA treated in UC mice could significantly increase the expressions of ZO‐1 and Occludin proteins, and these proteins were present along the inner layer of the columnar epithelium of colon tissue (Figure 2F,G). Besides, after GA treatment in UC mice, an obvious increased in the expression of mucin‐2 (Muc‐2) was also observed (Figure 2F,G). Interesting, the cytokine levels (including IL‐1β, IL‐6, IL‐17, and TNF‐α) were positively related to DAI score and histology score, and a negative correlation among cytokines level, body weight change, and colon length (Figure 2H). Meanwhile, we also found that GA treatment was negatively related to cytokines levels, DAI score and histology score, and positively related to intestinal barrier function, body weight change, and colon length (Figure 2H). Collectively, these results indicated that GA could modulate intestinal mucosal inflammation and protect against intestinal barrier function in DSS‐induced UC mice.

### 2.3 GA Inhibited Inflammation and Rescued Intestinal Barrier Integrity in Intestinal Organoids‐Induced by DSS

**Figure 3:**
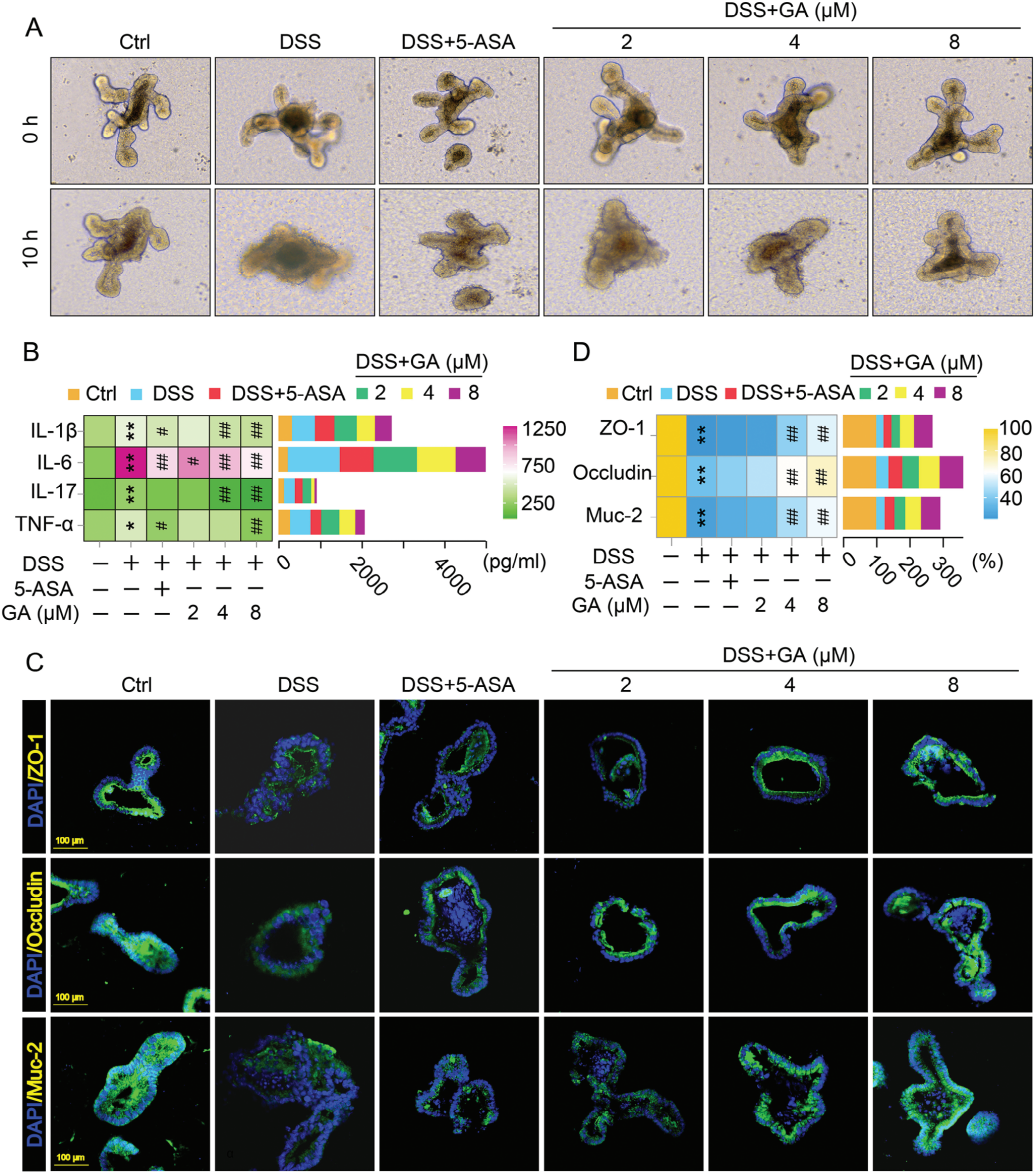
GA suppressed inflammation and protected against intestinal barrier dysfunction to attenuate DSS‐stimulated organoids damage. A) The morphological change of intestinal organoids in DSS stimulation (*n* = 4). B) The levels of pro‐inflammatory cytokines (IL‐1β, IL‐6, IL‐17, and TNF‐α) in the supernatant (*n* = 4). C) Representative immunofluorescence images of ZO‐1, Occludin and Muc‐2 in intestinal organoids (*n* = 4). D) The fluorescence intensities of ZO‐1, Occludin, and Muc‐2 in DSS‐stimulated organoids (*n* = 4). All data are expressed as mean ± S.E.M. ^*^P < 0.05 and ^**^P < 0.01 compared with the control group (Ctrl), *^#^P* < 0.05 and ^##^ *P* < 0.01 compared with the DSS group.

To further confirm the effect of GA on inhibiting inflammation and rescuing intestinal barrier integrity, DSS‐induced intestinal organoids damage model was used. We observed that a 10 h co‐incubation with 1–8 µm GA dose‐dependently increased the viability of intestinal organoids (Figure S2A, Supporting Information). However, when the concentration of GA is greater than 8 µm, GA significantly inhibited the vitality of intestinal organoids as compared to 8 µm GA (Figure S2A, Supporting Information). Thereafter, intestinal organoids were co‐incubated with DSS (0.1%) and GA (1–32 µm) for 10 h. We found that GA dose‐dependently inhibited the damage of intestinal organoids‐caused by DSS in the dose range of 2–8 µm (Figure S2B, Supporting Information). Therefore, we selected the GA concentrations of 2, 4, and 8 µm as experimental doses to explore the effect of GA on regulating inflammation and barrier function in DSS‐induced intestinal organoid model. DSS‐exposed to the organoids caused 3D culture organoids disintegration and gut cell apoptosis (**Figure** **3**A). Importantly, GA significantly inhibited organoids disintegration and reduced apoptosis (Figure 3A), indicating that GA protected against DSS‐induced organoids damage. Similar results for the anti‐inflammatory effect of GA also were observed in DSS‐induced organoid model, DSS intervened to organoids could significantly increase the secretion of IL‐1β, IL‐6, IL‐17, and TNF‐α cytokines, which were dose‐dependently inhibited after treatment with GA (Figure 3B). Additionally, the immunofluorescence analysis indicated that the distribution and fluorescence intensity of the tight junction proteins (ZO‐1, Occludin) and Muc‐2 were dramatically improved by GA treatment in DSS‐induced organoids (Figure 3C,D). Collectively, these results indicated that GA treatment alleviated inflammation and restores the tight junction damage in UC model mice and DSS‐induced organoids.

### 2.4 GA Regulated IL‐17RA Signaling to Alleviate Inflammation and Intestinal Barrier Damage in DSS‐Induced UC Model

**Figure 4:**
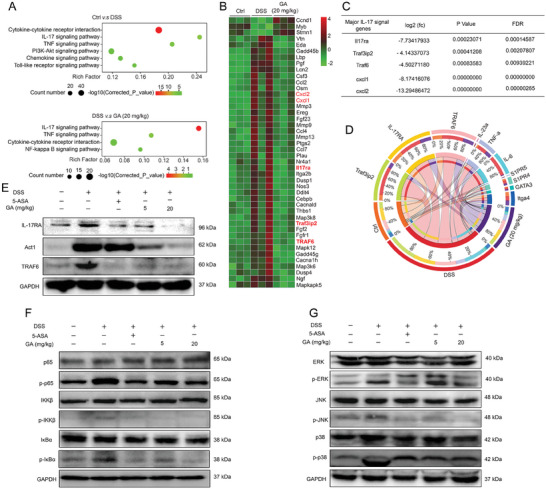
Effect of GA on regulating IL‐17RA signaling and downstream signaling in DSS‐induced colitis mice. A) KEGG analysis. B) Heatmap of IL‐17RA signaling and downstream signaling genes. C) The fold change (fc), p value and false discovery rate (FDR) of IL‐17 signaling key genes. D) Circos represents different genes and different groups, the innermost lines showed the connections between the genes and groups. The thickness of the lines indicates the expressions levels of genes. E–G) Western blot analysis for IL‐17 signaling and downstream signaling proteins in colonic tissues of colitis mice. All data are expressed as mean ± S.E.M (*n* = 3). ^*^P < 0.05 and ^**^P < 0.01 compared with the control group, *^#^P* < 0.05 and ^##^ *P* < 0.01 compared with the DSS group.

To investigate the mechanism of GA on alleviating DSS‐induced UC mice, we performed RNA‐seq analysis on colonic tissues among the control group, DSS model group and GA group. As shown in **Figure** **4**A, kyoto encyclopedia of genes and genomes (KEGG) analysis indicated that pathways such as cytokine‐cytokine receptor interaction, IL‐17 signaling pathway, TNF signaling pathway, chemokine signaling pathway and toll‐like receptor signaling pathway were significantly affected between the control group and DSS group. Interestingly, GA‐administrated UC mice markedly intervened the expressions of IL‐17 signaling pathway, TNF signaling pathway, cytokine‐cytokine receptor interaction and NF‐kappa B signaling pathway in colonic tissues (Figure 4A).

Since TNF signaling pathway, chemokine signaling pathway and NF‐kappa B signaling pathway are regulated by IL‐17 signaling.^[^ ^31^ ^]^ We sought to analyze the detailed IL‐17 signaling‐related genes expressions. We found that DSS exposed to mice memorably activated IL‐17RA signaling and downstream related genes expressions in colonic tissues (Figure 4B). Conversely, GA‐treated UC mice significantly suppressed the expressions of IL‐17RA signaling (Figure 4C) and downstream related genes (Figure 4B) expressions. Importantly, according to circos and heat maps analysis, in the transcriptome result, we found that GA is more likely to affect the expression of IL‐17RA signaling gene (over 81%) as compared to current IBD targets (Figure 4D), indicating that GA may alleviate UC by regulating the IL‐17RA signaling. To further demonstrate the importance of IL‐17RA signaling to UC, mantel test analysis and corr_heatmap analysis were performed. As expected, mantel test analysis revealed that key genes (including IL‐17RA, Act1, and TRAF6) in IL‐17RA signaling have significant effect on UC clinical symptoms, colon injury, pro‐inflammatory cytokines expression and intestinal barrier function (Figure S3A, Supporting Information). Meanwhile, corr_heatmap analysis also showed that the expression of IL‐17RA, Act1, and TRAF6 proteins have strong negative correlation with body weight, colon length, ZO‐1, Occludin and Muc‐2 in colitis mice, and a significant positive correlation with DAI score, histopathological score and pro‐inflammatory cytokines (Figure S3B, Supporting Information). In addition, we also established an alluvial plot to illustrate the potential relationships among IL‐17RA signaling, inflammation, intestinal barrier function and UC clinical symptoms. The key genes in IL‐17RA signaling showed significant correlations with pro‐inflammation cytokines (IL‐1β, IL‐6, IL‐17, and TNF‐α) and intestinal barrier function to mediate the progression of UC (Figure S3C, Supporting Information). Collectively, these results suggested that IL‐17RA signaling plays an important role in the pathological process of UC.

To confirm the effect of GA on regulating IL‐17RA signaling and downstream signaling, the proteins levels of IL‐17RA, Act1, TRAF6, p‐p65, p‐IKKβ, p‐IκBα, p‐ERK, p‐JNK, p‐p38 in colonic tissues were detected. As shown in Figure 4E–G, we observed that GA treatment in UC mice was conspicuously inhibited the activation of IL‐17RA signaling (e.g. IL‐17RA, Act1, TRAF6), NF‐κB signaling (e.g. p‐p65, p‐IKKβ, p‐IκBα) and MAPK signaling (e.g. p‐ERK, p‐JNK, p‐p38) in colonic tissues (Figure S4A–I, Supporting Information). Overall, these results indicated that IL‐17RA signaling is involved in the progression of UC, and the effect of GA on treating UC is closely related to inhibit the activation of IL‐17RA signaling.

### 2.5 GA Protected Against rIL‐17A‐Induced Organoid Damage via Inhibiting Inflammation and Restoring Intestinal Barrier Function

**Figure 5:**
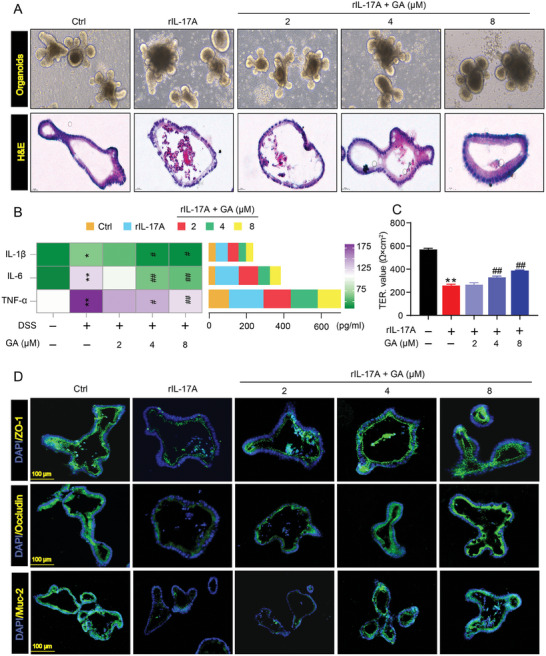
GA treatment alleviated rIL‐17A‐induced organoid damage by suppressing inflammation and protecting against intestinal barrier damage. A) Representative images of organoids treated with rIL‐17A and GA, and representative H&E staining of organoids. B) IL‐1β, IL‐6 and TNF‐α levels in the supernatant from rIL‐17A‐stimulated organoids with or without GA. C) The TER value of NCM460 cells. D) Representative fluorescent images of ZO‐1, Occludin, and Muc‐2 in the organoids. All data are expressed as mean ± S.E.M (*n* = 4). ^*^P < 0.05 and ^**^P < 0.01 compared with the control group, *^#^P* < 0.05 and ^##^ *P* < 0.01 compared with the rIL‐17A group.

Given that GA maybe inhibit IL‐17RA signaling to alleviate DSS‐induced UC mice, we established a rIL‐17A‐induced intestinal organoid model to mimic gut damage, and further explore the effect of GA on regulating inflammation and intestinal barrier function (Figure S5A, Supporting Information). The rIL‐17A exposure induced the disintegration of organoid and increased the apoptosis of enterocytes (**Figure** **5**A), this result is consistent with previous report.^[^ ^32^ ^]^ H&E analysis also revealed that a disorganized epithelium occurred in rIL‐17A‐treated organoid (Figure 5A). Importantly, GA treatment significantly mitigated rIL‐17A‐induced organoid damage evidenced by dose‐dependently inhibiting disintegration and apoptosis of organoids (Figure 5A). Additionally, we observed that GA treatment showed obvious epithelial and mesenchymal domains with an ordered arrangement by H&E staining (Figure 5A), indicating that GA has a significant protective effect against rIL‐17A‐induced organoid damage.

Since inflammation and intestinal barrier disorder are the important pathological features in UC patients and colitis animal model.^[^ ^27^, ^28^ ^]^ We next tested whether GA could diminish rIL‐17A‐induced organoid damage via inhibiting inflammation and remodeling intestinal barrier function. In line with the results of in vivo, we observed that GA intervened rIL‐17A‐stimulated organoid model could significantly blunt the release of pro‐inflammatory cytokines IL‐1β, IL‐6 and TNF‐α (Figure 5B). Meanwhile, similar results also were found in NCM460 cells with rIL‐17A stimulation, as shown by markedly reducing the expressions of pro‐inflammatory cytokines after GA treatment (Figure S5B–D, Supporting Information). To summarize, we evidenced that GA could inhibit inflammation to mitigate gut damage.

Subsequently, we further validated the effect of GA on improving intestinal barrier function. The transepithelial electrical resistance (TER) and FITC‐dextran staining were performed to monitor the cell membrane permeability after rIL‐17A combined with GA intervene. We found that GA treatment significantly increased the TER of rIL‐17A‐exposed NCM460 cells monolayers (Figure 5C). Consistently, fluorescent images indicated that GA treatment obviously decreased the expression of FITC‐dextran in rIL‐17A‐stimulated NCM460 cells (Figure S5E,F, Supporting Information). Notably, GA also revealed a weakening effect on the disruption of epithelial tight junction disruption as evidenced by increasing the expression of ZO‐1, Occludin and Muc‐2 proteins in the columnar epithelial lining of organoids (Figure 5D; Figure S5G, Supporting Information). Besides, after GA treatment in organoids, the levels of Tjp1, Occludin and Muc‐2 mRNA were also significantly increased (Figure S5H–J, Supporting Information). These results indicated that GA could rescue the destruction of epithelial tight junctions induced by rIL‐17A.

### 2.6 GA Alleviated rIL‐17A‐Induced Intestinal Organoid Injury via Suppressed IL‐17RA Signaling

**Figure 6:**
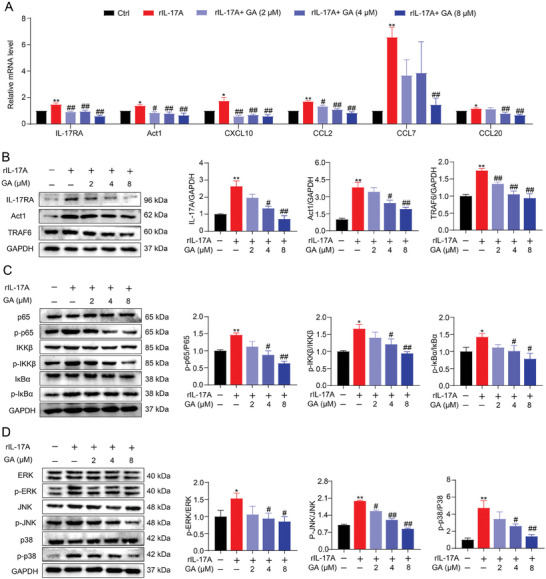
GA suppressed IL‐17 downstream signaling activation in rIL‐17A‐induced organoids. A) The genes levels of IL‐17 and its downstream chemokines were detected by RT‐PCR (*n* = 3‐5). B) The expression of IL‐17 signaling key proteins (IL‐17RA, Act1, and TRAF6) in the organoid was detected by immunoblotting analysis (*n* = 3). C) The expression of NF‐κB signaling proteins (p65, p‐p65, IKKβ, p‐IKKβ, IκBα, and p‐IκBα) in the organoid was examined by Western blot analysis (*n* = 3). D) The expression of MAPK signaling proteins (ERK, p‐ERK, JNK, p‐JNK, p38, and p‐p38) in the organoid was determined by Western blot analysis (*n* = 3). All data are expressed as mean ± S.E.M. ^*^ *P* < 0.05 and ^**^ *P* < 0.01 compared with the control group, ^#^ *P* < 0.05 and ^##^ *P* < 0.01 compared with the rIL‐17A group.

IL‐17RA signaling was reported to exacerbate mucosal inflammation and damage intestinal barrier in the progression of UC.^[^ ^33^, ^34^ ^]^ To further analyze the roles of IL‐17RA signaling in GA‐mediated protection, rIL‐17A‐stimulated intestinal organoid model was used. As expected, we found that GA treatment inhibited the expression of IL‐17 signal genes and downstream chemokines genes, including IL‐17RA, Act1, CXCL10, CCL2, CCL7 and CCL20 in rIL‐17A‐induced organoids in a dose dependent manner (**Figure** **6**A). Consistent with these results, we also observed that the expression of proteins, such as IL‐17RA, Act1, TRAF6, p‐65, p‐IKKβ, p‐IκBα, p‐ERK, p‐JNK, and p‐38 in rIL‐17A‐stimulated organoids down‐regulated by GA administration (Figure 6B–D). Overall, these results suggested that GA could block the activation of IL‐17RA and downstream signaling to protect against colon damage and epithelial tight junction disruption.

### 2.7 IL‐17RA was a Direct Target of GA

**Figure 7:**
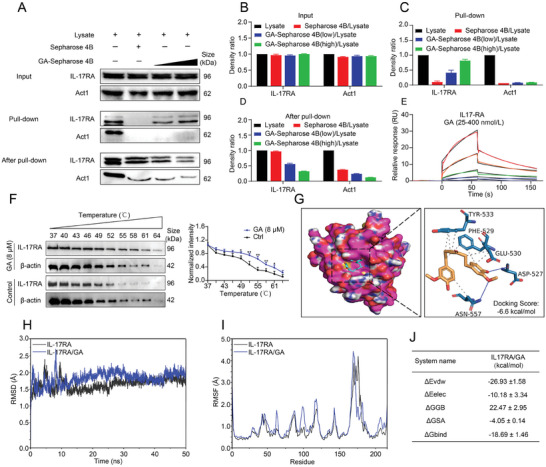
GA directly binds to the IL‐17RA. A–D) The binding of IL‐17RA and GA was determined using immunoblotting, and the density of proteins was analyzed in total (input), pull‐down and after pull‐down. E) The SPR analysis of the direct interaction between GA and IL‐17RA. F) Cellular thermal shift assay of IL‐17RA in NCM460 cells, cells treated with or without GA (8 µm) for 12 h. G) Docking analysis between GA and IL‐17RA protein (4NUX). H) The change of root mean square deviation (RMSD) with time in molecular dynamics simulation. I) Root Mean Square Fluctuation (RMSF) was calculated using molecular dynamics simulation trajectory. J) Binding free energies and energy components predicted by MM/GBSA. All data are expressed as mean ± S.E.M (*n* = 3). ^*^P < 0.05 and ^**^P < 0.01 compared with the control group.

According to the results in vivo and in vitro, we found that GA could markedly inhibit IL‐17RA signaling activation. However, the molecular target of GA on suppressing inflammation and improving barrier function still remained unclear. To elucidate that the regulated effect of GA on inflammation and barrier is closely related to IL‐17RA signaling, we performed a pull‐down assay to identify the potential molecular target of GA. We observed that GA physically binds to the active IL‐17RA protein, but not to Act1 protein (**Figure** **7**A–D), indicating that the direct interaction between GA and IL‐17RA protein. Consistently, SPR analysis also evidenced that GA (25–400 nmol L^−1^) directly interacted with IL‐17RA in a concentration‐dependent manner (Figure 7E). More importantly, CETSA analysis showed that GA markedly protected IL‐17RA protein from temperature‐dependent denaturation (Figure 7F). In addition, the docking prediction result revealed that GA has hydrophobic effect with IL‐17RA protein (PDB: 4NUX) residues, including ASN‐557, PHE‐592, TYR‐533, and Glu‐530. and GA compound formed two important hydrogen bonds with the residues of ASN‐557 and ASP‐527 of IL‐17RA, and the binding energy scores between IL17RA and GA is −6.6 kcal mol^−1^ (Figure 7G).

Because the molecular docking only offer the static state between compound and protein, so we further performed molecular dynamic stimulation to investigate the dynamic movement and stability of GA and IL‐17RA protein. The root mean square deviation (RMSD) values of IL‐17RA/GA and IL‐17RA have been stable at 1–2.5 Å in 0–50 ns (Figure 7H), indicating that there is no obvious conformational change between IL‐17RA/GA and IL‐17RA. Meanwhile, the root mean square fluctuation (RMSD) of the IL‐17RA/GA was relatively low, and most of the sequences on the protein has RMSFs below 2 Å (Figure 7I), indicating that the protein system is still relatively stable. Besides, the binding energies of IL‐17RA/GA compound was −18.69 ± 1.46 kcal mol^−1^ (Figure 7J). Overall, these results confirmed that GA directly binds to IL‐17RA, thereby suppressing IL‐17RA signaling to regulate inflammation and intestinal barrier function.

### 2.8 Effect of GA on Alleviating UC Depended on IL‐17RA Signaling

**Figure 8:**
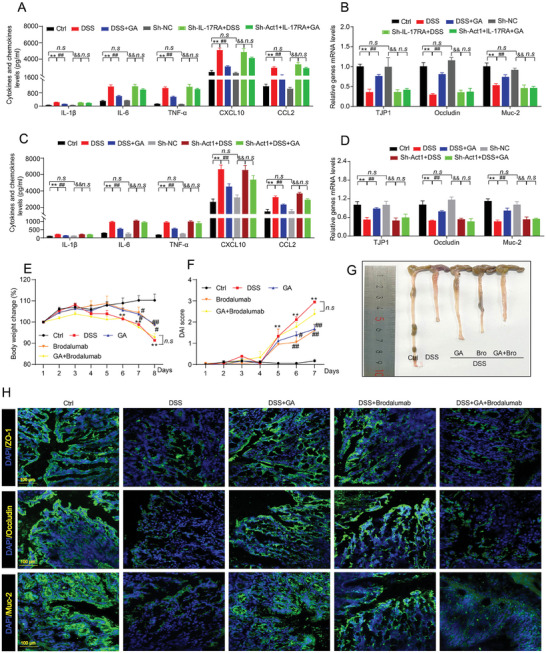
IL‐17 RA and downstream signaling mediated protective effect of GA on allevaiting UC. A,B) IL‐17RA was knocked down in intestinal organoid, then the organoids were stimulated with 0.01% DSS and treated with GA for 10 h. (A) The levels of pro‐inflammatory cytokines and chemokines (*n* = 3). (B) The mRNA expressions of TJP1, Occludin, and Muc‐2 (*n* = 3). C,D) Act1 was knocked down in organoids, then the organoids were stimulated with 0.01% DSS and treated with GA for 10 h. (C) The levels of pro‐inflammatory cytokines and chemokines (*n* = 3). (D) The mRNA expressions of TJP1, Occludin, and Muc‐2 (*n* = 3). E–K) C57BL/6 mice induced by 3% DSS for 7 days, and then the mice were orally administered with GA (20 mg kg^−1^, 7 days) or intraperitoneally injected with brodalumab (0.55 mg kg^−1^, 7 days), and the co‐treatment of GA and brodalumab in colitis mice was also performed in this study for 7 days. (E) The percentage of body weight change. (F) DAI score. (G) The representative image of colon length. (H) The representative fluorescent images of ZO‐1, Occludin and Muc‐2 in colonic tissues. All data are expressed as mean ± S.E.M (*n* = 6). ^*^P < 0.05 and ^**^P < 0.01 compared with the control group, *^#^P* < 0.05 and ^##^ *P* < 0.01 compared with the DSS group, *^&&^P* < 0.01 compared Sh‐NC group, *n.s* indicates not significant (*P* > 0.05).

To further confirm the effect of GA on modulating inflammation and intestinal barrier function thought IL‐17RA signaling, we first knockdown IL‐17RA using lentiviral expression plasmid on organoids (Figure S6A, Supporting Information). As shown in **Figure** **8**A, we found that IL‐17RA knockdown significantly diminished the effect of GA in DSS‐induced organoids model on inhibiting IL‐1β, IL‐6 and TNF‐α cytokines secretion, and GA mediated intestinal barrier function was also mainly impaired (Figure 8B). Based on this, we used the same method to further knockdown Act1 on organoids (Figure S6B, Supporting Information). Consistently, Act1 knockdown significantly abolished the effect of GA on inflammation and intestinal barrier function in DSS‐stimulated organoids (Figure 8C,D), suggesting that GA exerts its anti‐inflammation and restores intestinal barrier homeostasis mainly depending on inhibiting IL‐17RA/Act1 signaling.

In addition, to demonstrate the effect of GA on alleviating UC mouse model is dependent on IL‐17RA signaling, GA combined with brodalumab (an anti‐interleukin‐17‐receptor IgG2 monoclonal antibody) in treating DSS‐induced UC mouse model was performed (Figure S6C, Supporting Information). As expected, we observed that brodalumab alone can significantly improve the clinical symptoms (such as body weight loss, diarrhea and bloody stools) in DSS‐induced UC mouse model (Figure 8E,F), as well as inhibit colon injury and atrophy (Figure 8G; Figure S6D, Supporting Information). Conversely, the effect of GA on alleviating UC clinical symptoms (Figure 8E,F) and protecting against DSS‐induced colonic damage (such as inhibited colon atrophy, decreased inflammatory cell infiltration and crypt structure destruction in colonic tissues) was significantly emasculated after combined with brodalumab (Figure 8G; Figure S6D–F, Supporting Information). Furthermore, as compare to the GA group and brodalumab group, the co‐treatment of GA and brodalumab also did not show better effect on inhibiting pro‐inflammatory cytokines (such as IL‐1β, IL‐6, and TNF‐α) release and restoring intestinal barrier homeostasis in DSS‐induced UC mice (Figure 8H; Figure S6G–J, Supporting Information). Interestingly, these results were consistent with the co‐treatment of GA and ixekizumab (Taltz, an IL‐17A antagonist). The effect of GA on alleviating colitis was significantly impaired after co‐treatment with ixekizumab (Figure S7, Supporting Information). Taken together, these results indicated that the combination of GA and brodarumab or IL‐17A antagonist exist a competitive antagonistic effect on binding to IL‐17RA target, which may be result in a negative effect for GA treatment UC mice, indicating that the protective effect of GA against UC is primarily dependent on IL‐17RA signaling.

## 3 Discussion

UC is an inflammatory disorder of the colonic mucosa with an unknown etiology. Currently, there is an urgent need to investigate novel and effective therapeutic agents.^[^ ^22^, ^35^ ^]^ In this study, we first reported that GA treatment in DSS‐induced UC mice could significantly ameliorate body weight loss, diarrhea, bloody stools, colon atrophy, inflammatory cell infiltration, crypt damage, epithelial destruction, and intestinal barrier dysfunction in colon tissues. More importantly, we further confirmed that GA directly binds to the IL‐17RA active protein and suppresses IL‐17RA signaling and downstream inflammatory signaling (e.g., NF‐κB, MAPKs) activation to regulate inflammation and intestinal barrier function in vivo and in vitro. Collectively, our studies indicated that GA may be a potential novel agent for the treatment of UC.

As we all known, DSS‐induced UC models have similar clinical features and pathological changes to UC patients, including body weight loss, diarrhea, hematochezia, colonic diffuse inflammatory infiltration and intestinal barrier damage, etc, which have become an important tool to explore the pathogenesis of UC and study novel drugs for UC treatment.^[^ ^22^, ^36^ ^]^ GA is one of the major polyphenolic ingredients and isolated from *Zingiber officinale*. Previous studies have confirmed that GA has obvious anti‐inflammatory effect.^[^ ^19^ ^]^ However, whether GA can treat intestinal diseases (such as UC) caused by inflammation remains undetermined. To investigate the protective effect of GA on attenuating UC, DSS‐induced UC mouse model was performed. Our results revealed that GA treatment in UC mice showed dramatic therapeutic effects with the manifestation of significantly inhibiting body weight loss, diarrhea, hematochezia and alleviating colon damage. Additionally, GA was observed to markedly suppress AOM/DSS‐induced colonic tumorigenesis. This indicates that GA could protect against colitis and its associated tumor in mice.

Accumulated evidences suggest that inflammation and intestinal barrier dysfunction are not only the main pathological features of UC, but also the key targets to elucidate the pathogenesis of UC and explore the prevention and treatment drugs of UC.^[^ ^37^, ^38^, ^39^ ^]^ The intestinal epithelium shapes the largest interface of body with the external environment, it provides an important barrier to selectively limit the penetration of toxins and antigens via the intestinal mucous membrane, while also participating in nutrients and water absorption.^[^ ^40^, ^41^ ^]^ Tight junctions (TJs) as an important physical barrier in the gut, its core structure and function are mainly dependent on transmembrane proteins (such as Occludin) and skeleton connexin (such as ZO‐1) to regulate the function of gate, which play a crucial role in maintaining the homeostasis of the intestinal barrier and regulating intestinal permeability.^[^ ^42^, ^43^ ^]^ Except for physical barrier, the mucus barrier also protects gut from pathogens and symbiotic bacteria‐induced inflammation. For example, Muc‐2, a major colonic mucin, which is secreted by goblet cells and devotes to maintaining the integrity of the intestinal barrier and inhibiting inflammatory stimulation for intestine.^[^ ^44^ ^]^ However, under pathological conditions, the obvious loss of TJs proteins (including ZO‐1, Occludin, and Muc‐2) can impair intestinal barrier function and induce intestinal inflammatory responses.^[^ ^30^, ^45^ ^]^ Moreover, ZO‐1 or Occludin or Muc‐2 knockdown mice have been evidenced to increase the risk of gut inflammation and block mucosal repair, indicating that intestinal barrier involves in the occurrence and development of UC.^[^ ^46^, ^47^ ^]^ Besides, the crosstalk between intestinal barrier and intestinal immunity plays an important role in the pathogenesis of UC. The abnormal change of intestinal barrier can cause the aggressive behavior of intestinal immunity, and further lead to a large release of pro‐inflammatory factors including IL‐1β, IL‐6, and TNF‐α to aggravate colon injury.^[^ ^48^ ^]^ Indeed, in UC, the severe inflammatory responses in colon tissue are significantly negatively correlated with intestinal barrier function and increased the risk of CRC. Consistently, the inhibition of gut inflammation can significantly protect against intestinal barrier damage and inhibit CRC.^[^ ^49^, ^50^ ^]^ Notably, our studies showed that GA treatment can significantly inhibit the expression of pro‐inflammatory cytokines IL‐1β, IL‐6, and TNF‐α in UC mice and intestinal organoids. Moreover, GA protected against the increase of intestinal permeability and loss of intestinal barrier integrity induced by DSS or rIL‐17A stimulation in vivo and in vitro. These results indicated that the protective effect of GA on ameliorating UC may be closely related to inhibit inflammation and maintain intestinal barrier integrity.

Although previous studies have confirmed that GA can regulate inflammatory signaling to inhibit the expression of pro‐inflammation cytokines, and treat inflammatory related diseases,^[^ ^19^ ^]^ the molecular mechanism of GA in colitis is still unclear. To explore the underlying mechanism of GA on protecting against DSS‐induced colitis, RNA‐seq analysis was performed. We observed that the expressions of IL‐17RA signaling relative genes were significantly increased after DSS‐administration. However, GA treatment could significantly down‐regulate IL‐17RA signaling genes expressions. Consistent with the results of RNA seq, we further found that GA could markedly suppress the proteins expressions of IL‐17RA signaling and downstream signaling (including NF‐κB, MAPK) in colitis mice and intestinal organoids. Indeed, IL‐17RA signaling involves in the pathogenesis of UC and has been linked to promote the expression of pro‐inflammatory cytokines (including IL‐1β, IL‐6, and TNF‐α etc) and chemokines (such as CXCL1, CCL2, CXCL2, and CCL20 etc), as well as lead to intestinal barrier disruption.^[^ ^12^, ^33^ ^]^ Consistently, in this study, the correlation analysis results showed that IL‐17RA signaling key genes were correlated with colitis clinic features, inflammation and intestinal barrier, which revealed that IL‐17RA signaling play a vital role in the development of UC. To further explore the effect between GA and IL‐17RA signaling, by using pull down, plasmon resonance analysis and molecular dynamic stimulation, we found that GA could directly bind to IL‐17RA active protein, and then exert its inhibiting effect on regulating NF‐κB/MAPK pathway. More importantly, in IL‐17RA gene knockdown organoids, we observed that the effects of GA on alleviating inflammation and restoring intestinal barrier were significantly eliminated. Meanwhile, similar results also were found in Act1 gene knockdown organoids. Besides, we also observed that concurrent supplementation with GA and IL‐17R lgG2 monoclonal antibody did not produce the synergistical effect on alleviating DSS‐induced mouse model. Collectively, these results indicated that GA could target IL‐17RA signaling to inhibit inflammation and restore intestinal barrier function, and then protect against DSS‐induced colitis.

In summary, in this study, we first demonstrated that oral administration of GA can alleviate DSS‐induced colitis mouse model by inhibiting inflammation and protecting intestinal mucosal barrier. We further identified that GA directly binds to IL‐17RA active protein, which inhibits downstream signaling to protect against UC, suggesting that GA could be a novel potential option for UC treatment.

## 4 Experimental Section

### Reagents

Dextran sulfate sodium (DSS, MW 36–50 kDa) and AOM were purchased from MP Biomedicals (Irvine, CA, USA). Gingerenone A (SC‐490979, Figure S1A, Supporting Information) and primary antibody against Act1 (SC‐100647) were obtained from Santa Cruz Biotechnology (San Diego, CA, USA). The primary antibodies of GAPDH (40 493), TRAF6 (32 102), p65 (48 676), p‐p65 (11 010), IκBα (48 695), p‐IκBα (48 695), IKKβ (27 236), p‐IKKβ (11 929), p38 (49 379), p‐p38 (11 581), JNK (48 615), p‐JNK (13 371), ERK (40 901), p‐ERK (13 326) were obtained from Signalway Antibody (SAB, Pearland, TX, USA). The primary antibody against NIMP‐R14 (ab2557) and muc‐2 (ab272692) was obtained from Abcam (Cambridge, MA, USA). Anti‐ZO‐1 (13 663) was purchased from Cell signaling Technology (Danvers, MA, USA). Anti‐IL‐17RA (DF3602) and anti‐Occludin (DF7504) were obtained from Affinity Bioreagents (Cincinnati, OH, USA). Mouse IL‐1β, IL‐6, TNF‐α and IL‐17A ELISA kits were purchased form Lianke Biology (Hanzhou, China). Sepharose 4B and FITC‐dextran (MW: 4000) were obtained from Sigma–Aldrich (St. Louis, MO, USA). Recombine mouse IL‐17A (421‐ML‐010) were obtained from R&D (Minnesota, USA). AG RNAex Pro Reagent (AG21102), Evo M‐MLV RT Mix kit (AG11728) and SYBR^@^ Green Premix Pro Taq HS qPCR kit (AG11701) were Accurate Biotechnology (Hunan) Co. LTD (Changsha, China). Y27632 (S83435) and SB431542 (S80607) were purchased from Shanghai yuanye Bio‐Technology (Shanghai, China). G418 (GC17427) was purchased from GLPBIO (Montclair, CA, USA). Hygromycin B was obtained from Coolaber (Beijing, China). Matrigel (40188ES10) was purchased from Yeasen (Shanghai, China). Lentiviral expression plasmids for sh‐IL‐17RA and sh‐TRAF3IP2 (known as Act1) were purchased from HanBio Co., Ltd (Shanghai, China). Bicinchonininc acid (BCA) protein assay kit was obtained from Meilunbio (Dalian, China). rodalumab (HY‐P9925) was purchased from MedChemExpress (Shanghai, China). Ixekizumab (also known as Taltz, D378793CA) was obtained from Lilly Trading Co., Ltd (Suzhou, China).

### Experimental Animals

Male C57BL/6 mice (6–8 weeks of age, 22 ± 2 g) were purchased from the Laboratory Animal Center of Guangzhou University of Chinese Medicine (SYXK 2018‐0085). All mice were maintained under a specific pathogen free (SPF) environment with light/dark cycling for 12 h, controlled temperature (22–24 °C), humidity (50–60%), and free accessed to water and food. All animal experiments were conducted in accordance with the Guidelines for Humane Use of Animals issued by the National Institutes of Health and approved by the Animal Ethics Committee of Guangzhou University of Chinese Medicine (No. 20 230 213 014).

### Ulcerative Colitis Model Establishment and Treatment

Mice were acclimatized for 7 days in SPF environment, and then all mice were randomly divided into 5 groups (*n* = 12 per group): control group (Ctrl), DSS group (DSS), DSS + 5‐aminosalicylic acid positive group (5‐ASA, 100 mg kg^−1^), and DSS + Gingerenone A groups (GA, 5 and 20 mg kg^−1^). The UC model of mice was established by free drinking water of 3% DSS (W/V) for 7 days, while mice in control group were given distilled water freely. Mice in control group and DSS group were given sterile water (10 mL kg^−1^, i.g), 5‐ASA group and GA groups (The optimal dose range of GA was selected according to the results of the preliminary experiment) mice were given corresponding concentrations of drugs once a day (Figure S1B, Supporting Information). All mice were euthanized by inhaling isoflurane on day 8. The blood samples and colon tissues were quickly collected for the subsequent analysis.

CAC mouse model was established using AOM and DSS as described previously.^[^ ^51^ ^]^ All mice were randomly divided into 5 groups, including control group, AOM/DSS group, AOM/DSS+5‐Fu group and AOM/DSS+Gingerenone A groups (GA, 5 and 20 mg kg^−1^). The AOM/DSS group, AOM/DSS+5‐Fu group and AOM/DSS+GA groups were injected with AOM (10 mg kg^−1^. i.p.). One week later, the drinking water for the mice in the AOM/DSS, AOM/DSS+5‐Fu group and AOM/DSS+GA groups were replaced with 2.5% DSS (w/v) in distilled water for 7 days, and then the mice were supplemented with distilled water for 10 days to recover. This cycle was duplicated 3 times. The mice in control group and AOM/DSS group were administered sterile water (10 mL kg^−1^. p.o.) once a day, 5‐Fu (20 mg kg^−1^. i.p.) and GA (5 and 20 mg kg^−1^. p.o.) were administrated in CRC model mice, respectively (Figure S1G, Supporting Information). At the end of the experiment, all mice were euthanized for the subsequent analysis.

### Disease Activity Index Analysis

DAI was determined according to body weight loss, diarrhea and hematochezia. The evaluation criterion for body weight loss, diarrhea and hematochezia was described as previously reported^[^ ^36^ ^]^: body weight loss (0 score, none, 1 score, 1−5%, 2 score, 6−10%, 3 score, 11–20%, 4 score, >20%), diarrhea (0 score, normal feces, 1 score, loose but formed, 2 score, loose without formed, 3 score, very loose, 4 score, severe diarrhea), hematochezia (0 score, negative, 1 score, weakly positive, 2 score, positive, 3 score, obviously positive, 4 score, massive rectal bleeding).

### RNA Sequencing Analysis

According to the manufacturer's instructions, total RNAs from colon tissues were isolated using trizol reagent (Life Technologies, Carlsbad, CA). mRNA’ amount and quantity in the total RNA were measured by Nanodrop 2000. RNA‐seq libraries were prepared and sequenced on the Illumina sequencing platform by Wuhan Metware Biotechnology Co., Ltd (Guangzhou, China). The standard for FDR below 0.05 and absolute fold change ≥1.5 was performed to analyze the significantly different genes, and the OmicShare tool was used for gene expression heat map, KEGG pathway analysis.

### Cell Culture and the Preparation of Intestinal Organoid Medium

NCM460 cells were purchased from the iCell Bioscience Inc (Shanghai, China) and cultured in RPMI 1640 medium containing 10% FBS and 1% penicillin and streptomycin. The cells were maintained in an incubator at 37 °C, 5% CO_2_ and 95% air.

L‐WRN cells were obtained from Meisen Chinese Tissue Culture Collections (CTCC‐001‐0253, Zhejiang, China), transfecting L‐Wnt3A with R‐Spondin3 and Noggin co‐expression vectors. L‐WRN can be steadily cloned in culture medium containing G418 (0.5 mg mL^−1^) and hygromycin B (0.5 mg mL^−1^), and secrete Wnt3A, R‐Spondin3 and Noggin factors into culture medium.

The intestinal organoids culture medium that contained 20% FBS and recovered conditioned medium from L‐WRN cells was obtained. In brief, L‐WRN cells were maintained in DMEM/F12 supplemented with 20% FBS, penicillin (100 µg mL^−1^), streptomycin (100 µg mL^−1^) and L‐glutamine (2 mm). The medium was collected and diluted 1:1 with the unused medium to form the intestine organoids basic medium. Gentamicin (10 µm), Y27632 (10 µm) and SB431542 (10 µm) were added into the medium to grow intestinal organoids.

### Crypt Isolation and Intestinal Organoid Culture

The method of crypt isolation from small intestines in mice was performed as previously reported with slight modification.^[^ ^52^ ^]^ Briefly, after euthanizing mice and collecting the intestines, the intestines were opened lengthwise and rinsed with pre‐cooled D‐PBS. The contents of the cavity and the villous structure were scraped with glass slide. To separate the crypts, the intestine was cut into pieces (2 mm) and incubated in EDTA (2.5 mm) at 4 °C for 30 min with a slight eddy current. After cleaning with pre‐cooled D‐PBS, the crypts were released by shaking manually for 3 min. A 70 µm filter was used to collect the supernatant, and the remaining tissue pieces were re‐suspended in pre‐cooled PBS, this cycle was repeated three times, and then the supernatant was centrifuged (290 g, 4 °C, 5 min) to obtain the crypts. After, the crypts was suspended in pre‐cooled D‐PBS and centrifuged at 200 g for 5 min at 4 °C. About 200 crypt globules were re‐suspended in cold organoids culture medium and matrigel (ratio = 1:1) and further induced the growth of intestinal organoids for 7–10 days on 24‐well culture plates (NEST, Wuxi, China).

### MTT Analysis

The appropriate number of organoids were plated on 96‐well culture plates for 5–7 days. After the organoids were treated with different concentrations GA (1‐32 µm) and stimulated with or without recombine mouse IL‐17A (100 ng mL^−1^) for 24 h. After, 10 µL MTT (5 mg mL^−1^) was added in each well and further incubated for 4 h at 37 °C, until the purple sedimets were found. The supernatant was removed and added 150 µL dimethylsulfoxide to each well. The optical density was measured using microplate reader at 490 nm.

### Histopathological Analysis

In order to observe the pathological changes, the colon tissue and organoids were fixed in 4% formalin solution for 48 h, then dehydrated and embedded in paraffin. The tissue sections were sliced into 4 µm, dewaxed with xylene, hydrated with a series of gradient concentrations of alcohol, and then stained with hematoxylin and eosin (H&E). Finally, the pathological changes of specimens were blindly analyzed under the light microscope. In addition, the colon tissues were scored according to the previous report scoring system^[^ ^53^ ^]^: normal (0 score); Lymphocyte infiltration was less than 10% (1 score); Lymphocyte infiltration was 10–25% lower (2 score); 25 to 50% moderate lymphocyte infiltration, increased vascular density and intestinal wall thickness (3 score); Lymphocyte infiltration was greater than 50%, goblet cells were reduced, and mucosal degeneration (4 score).

### Enzyme Linked Immunosorbent Assay

The levels of IL‐1β, IL‐6, IL‐17A, and TNF‐α in tissue homogenates or organoid culture supernatant were measured using ELISA kits according to the manufacturer's instruction and analyzed in microplate reader at 450 nm.^[^ ^54^ ^]^ Meanwhile, the content of total proteins in tissue homogenates was detected by BCA protein assay kit (Meilunbio, Shanghai, China) to normalize the expressions of cytokines.

### Real‐Time PCR Analysis

Total RNA was extracted from intestinal organoids using Trizol (Accurate Biotechnology, China) according to the manufacturer's instruction, and the concentration and quality of RNA were analyzed using Nanodrop 2000. After, the RNA was reverse‐transcribed into cDNA with Evo M‐MLV RT Mix kit (Accurate Biotechnology, Hunan, China). RT‐qPCR was performed using the CFX96 Real‐Time PCR Detection System (Bio‐Rad, USA). The relative expression levels of target genes were normalized with β‐actin (as internal reference), which were determined by 2^−ΔΔCt^. The mouse primer sequences were designed and listed in Table S1 (Supporting Information).

### Immunohistochemistry

Specimen slides were dewaxed with xylene, hydrated with gradient ethanol and repaired the antigens by citrate buffer (0.01 mol L^−1^) in the microwave. All slides were blocked with goat serum (3%) and incubated with primary antibody NIMP‐R14 (1:200) overnight at 4 °C. After the sections were incubated with broad‐spectrum secondary antibody for 2 h at room temperature, the immunoreactivity of target proteins were then detected using diaminobenzidine (DAB) kit (CST, USA), and the sections were counterstained with hematoxylin and dehydrated. Finally, pictures were obtain using the light microscope (Olympus, Japan), and the expressions of positive proteins were counted using Image J software (Wayen Rasband, NIH, USA).

### Immunofluorescence

Immunofluorescence analysis was performed on colon tissues and intestinal organoids as previous description.^[^ ^55^ ^]^ Briefly, sections were fixed with pre‐cooled acetone for 15 min, and then permeabilizd with 3% Triton X‐100. The sections were blocked with 1% BSA for 30 min at room temperature. After, sections were washed with PBS and incubated with primary antibodies including ZO‐1 (1:250), Occludin (1:250) and muc‐2 (1:250) overnight at 4 °C. On the second day, fluorescent secondary antibody was added and incubated for 2 h in room temperature, and the nuclei were stained with 4′,6‐diamidino‐2‐phenylindole (DAPI) reagent. Finally, the samples were imaged using a Zeiss fluorescence microscope (LSM 800, Germany).

### Western Blot

The colon tissue and intestinal organoids were lysed in RIPA buffer containing cocktail of protease and phosphatase inhibitors. The concentration of total proteins was determined using the BCA protein assay kit (Meilunbio, Dalian, China). Proteins were separated using electrophoresis in a 8% polyacrylamide gel and transferred to PVDF membranes. The membranes were blocked with 5% non‐fat milk for 2 h at room temperature, and then incubated with primary antibodies overnight at 4 °C. After rinsing with TBST three times, the membranes incubated with the secondary antibodies for 2 h at room temperature. The immunoreactive of target proteins were tested with the immobilon Western chemiluminescent HRP substrate kit (Millipore, USA) and captured using gel imaging system (Tanon, Shanghai, China). The relative expressions of target proteins were normalized to GAPDH.

### Molecular Docking

The 3D structure of human IL‐17RA protein (PDB ID: 4NUX) were obtained from the Protein Data Bank (https://www.rcsb.org/). The 3D structure of the small molecule GA was downloaded from the PubChem database (https://pubchem.ncbi.nlm.nih.gov/). Prior to the docking, PyMol 2.5.4 was used to treat with the receptor protein to remove water molecules, salt ions and small molecules. Next, autodock vina software (version: 1.1.2) was used to analyze molecular docking between GA and IL‐17RA, and the parameters for docking maintained at the default setting. The free binding energy was obtained as an indicator to reflect the binding, and the docking conformation with the highest output score was considered to be the binding conformation for subsequent molecular dynamics simulations.

### Molecular Dynamics Simulation

The GA and IL‐17RA protein complex was obtained from molecular docking and used as the initial structure for whole‐atom molecular dynamics simulation using AMBER 18 software. Before simulation, the charge of GA small molecules was calculated using the antechamber module and the Hartree‐FocK (HF) SCF/6‐31G* of Gaussian 09 software. After that, the GA and IL‐17RA protein were described by the GAFF2 small molecule force field and the ff14SB protein force field, respectively. LEaP module was performed to add hydrogen atoms and a truncated octahedral TIP3P solvent box was added at the distance of system 10 Å, and the charges balance of the system was maintained by adding Na^+^/Cl^−^. Before simulation, the energy of the system was optimized, and AMBER 18 software was used to conduct molecular dynamics simulation.

### MM/GBSA Combined Free Energy Analysis

MM/GBSA method was performed to calculate the binding free energies between proteins and ligands using the MD locus of 45–50 ns. The calculation method as described previously.^[^ ^56^ ^]^

### Surface Plasmon Resonance Analysis

Biacore 8K system (Cytiva, Marlborough, MA, USA) was performed to analyze the direct interaction between GA and IL‐17RA. IL‐17RA recombinant protein was immobilized on Series S Sensor Chip CM 5 (GE Healthcare Life, Chicago, USA) in accordance to the manufacturer's instruction. After that, different concentrations of GA (25–400 nmol L^−1^) were diluted in running buffer and injected to the system as the analyte. The parameters for SPR were performed as follows: flow rate, 30 µL min^−1^; association time, 60 s; dissociation time 90 s; temperature, 25 °C. Finally, the interaction parameters (such as *K_a_*, *K_d_* and *K_D_*) were obtained using Biacore evaluation software (Version 2.0).

### Pull‐Down Assay

Sepharose 4B freeze‐dried powder was activated in 1 mm HCl. GA was mixed with activated Sepharose 4B in coupling buffer (0.1 m NaHCO3 pH 8.3, 0.5 m NaCl) and rotated at 4 °C overnight. Then coupling buffer was replaced with 0.1 m Tris‐HCl buffer, and rotated again at 4 °C overnight. The GA‐Sepharose 4B compound was then washed with 0.1 m acetate buffer (pH 4.0) containing 0.5 m NaCl, followed by a second wash with 0.5 m NaCl. Proteins from NCM460 cells were incubated with GA‐Sepharose 4B beads or uncoupled Sepharose 4B beads in reaction buffer (50 mm Tris pH 7.5, 5 mm EDTA, 150 mm NaCl, 1 mm DTT, 0.01% Nonidet P‐40, 2 mg mL^−1^ bovine serum albumin, 0.02 mm PMSF) and rotated overnight at 4 °C. The beads were then washed with RIPA buffer and boiled for 8 min. Proteins bound to GA‐Sepharose 4B were analyzed by Western blot.

### Cellular Thermal Shift Assay

NCM460 cells (1×10^6^ per well) were seeded into culture dish. Cells were treated with or without GA (8 µm) for 12 h. Next, cells were collected and resuspended in PBS. The cells were heated for 3 min to 37, 40, 43, 46, 49, 52, 55, 58, 61 or 64 °C followed by 3 cycles of cooling at 4 °C. The soluble component were collected by centrifugation (15 000 g, 10 min) and analyzed by Western blot.

### TER Measurement

NCM460 cells were grown in 24‐well transwell chambers (Costar, size 0.4 µm) for 21 days to achieve a steady state of TER (more than 500 Ω*cm^2^). Cells were treated with GA (2, 4 and 8 µm) and stimulated with rIL‐17A for 48 h. After, TER was measured using an epithelial volt ohm meter (Millipore, MA, USA), and then the value of TER were determined by subtracting the blank filter and multiplying by the filter surface area.

### Permeability Assay

FITC‐dextran permeability assay in mouse. FITC‐dextran (MW: 4000) reagent was used to assay the intestinal permeability in colitis mouse model as previous reported. Briefly, all mice were forbidden food and water for 4 h, and then mice were gavage fed subsequently with FITC‐dextran solution (500 µg g^−1^). After 4 h, mice were euthanized and serum was collected. The concentration of FITC‐dextran in serum was detected at 480 nm excitation and 520 nm emission using SpectraMax I3X fluorescence microplate (Molecular Devices, USA).

### FITC‐Dextran Assay in Cell

NCM460 cells were seeded in laser confocal culture dish. After 12 h, cells were treated with different concentration of GA (2, 4 and 8 µm) and stimulated with rIL‐17A for 24 h. After, NCM460 cells were washed by PBS three times and further incubated with FITC‐dextran (MW: 4000) for 4 h. Cells were fixed in formaldehyde (4%) for 15 min at room temperature, washed, cells were stained with DAPI. Finally, the fluorescence were detected using Zeiss fluorescence microscope (LSM 800, Germany).

### Lentiviral Infection in Intestinal Organoid

The method of lentiviral infection in intestinal organoids was performed as described by Takahashi's report.^[^ ^57^ ^]^ These plasmids of lentiviral vector (pHBLV‐U6‐MCS‐CMV‐ZsGreen‐PGK‐PURO‐IL‐17RA or pHBLV‐U6‐MCS‐CMV‐ZsGreen‐PGK‐PURO‐TRAF3IP2 (Act1)) were purchased from Hanbio (Shanghai, China). Briefly, organoids cultured in matrigel (Corning, USA) were disrupted using 29G needle, and then cells were seeded in collagen I–coated 12‐well plates to induce 2D‐culture state and further cultured 4 days. 2D‐culture organoids were infected with lentiviral medium, which contained with polybrene (10 µg mL^−1^) by centrifugal method. After centrifugation, the cells were re‐cultured with fresh medium. Organoids were directly infected by incubating the virus solution with the recovered organoids in a 15 mL tube for 90 min. At the end of lentiviral infection, cells were re‐cultured in matrigel to restore the growth of organoids.

### The Co‐Treatment between GA and Brodalumab or Ixekizumab in DSS–Induced Colitis Mice

After adaptive feeding for 7 days, mice were randomly divided into 5 groups (*n* = 6 per group): control group (Ctrl), DSS group (DSS), DSS + GA group (20 mg kg^−1^), DSS+ brodalumab (2.7 mg kg^−1^) group (or DSS + ixekizumab (1.04 mg kg^−1^) group) and DSS + brodalumab (or ixekizumab) + GA group (20 mg kg^−1^). The UC model of mice was established by free drinking water of 3% DSS (W/V) for 7 days, while mice in control group were fed distilled water freely. Mice in control group and DSS group were given sterile water (10 mL kg^−1^, i. g). GA (20 mg kg^−1^) was administered by oral route into UC mice, and brodalumab or ixekizumab was delivered to mice by subcutaneous injection on 3 days apart. All mice were euthanized by inhaling isoflurane on day 8. The blood samples and colon tissues were quickly collected for the subsequent analysis.

### Statistical Analysis

Statistical analysis was performed using SPSS version 18.0 (Chicago, IL, USA) and plotted with Graphpad Prism software (Version 8.0.0, San Diego, California, USA). All data were expressed as mean ± standard error of mean (S.E.M). Comparisons between groups were performed by one‐way analysis of variance (ANOVA), with post‐hoc Tukey's test or Student's *t*‐test when appropriate. Mantel test was performed to analyze the correlation of two distance matrices. Pearson correlation was applied to compared the relationship among clinical symptoms of UC, inflammatory indexes, intestinal barrier and IL‐17 signal proteins. Relevant tools were used in the omicshare cloud platform (https://www.omicshare.com/) to create circos and heat maps. *P* values < 0.05 were considered as statistical significance.

## Conflict of Interest

The authors declare no conflict of interest.

## Author Contributions

J.L., W.D., and C.L. contributed equally to this work. Y.C., W.W., and H.T. designed the experiment and revised the final manuscript. J.L., W.D., C.L., Y.W., and C.C. conducted the experiments and analyzed the data. Y.X., S.H., and S.H. wrote the manuscript. C.L., S.H., and S.H. edited the manuscript. All authors read and approved the final manuscript.

## Supporting information

Supporting Information

## Data Availability

The data that support the findings of this study are available from the corresponding author upon reasonable request.
