# Supplementary material for: Gingerenone A Attenuates Ulcerative Colitis via Targeting IL‐17RA to Inhibit Inflammation and Restore Intestinal Barrier Function
Source: Adv Sci (Weinh). 2024 Apr 19;11(28):2400206. doi: 10.1002/advs.202400206 (PMC11267284; doi:10.1002/advs.202400206)
Supplement: Supplementary file 1 — Supporting Information [file ADVS-11-2400206-s001.pdf]

ADVANCED SCIENCE
Open Access

Supporting Information

for *Adv. Sci.*, DOI 10.1002/advs.202400206

Gingerenone A Attenuates Ulcerative Colitis via Targeting IL-17RA to Inhibit Inflammation and Restore Intestinal Barrier Function

Jian Liang, Weigang Dai, Chuanghui Liu, Yifan Wen, Chen Chen, Yifei Xu, Song Huang, Shaozhen Hou, Chun Li, Yongming Chen, Wei Wang* and Hailin Tang**

Supporting Information

Gingerenone A Attenuates Ulcerative Colitis via Targeting IL-17RA to Inhibit Inflammation and Restore Intestinal Barrier Function

Jian Liang[1, 2, 4, †], Weigang Dai[3, †], Chuanghui Liu[1, †], Yifan Wen[1], Chen Chen[1], Yifei Xu[5], Song Huang[1, 4], Shaozhen Hou[1], Chun Li[1], Yongming Chen[2,*,] Wei Wang[1, *], Hailin Tang[2,*]

Author Affiliations:

[1] School of Pharmaceutical Sciences, State Key Laboratory of Traditional Chinese Medicine Syndrome, Guangzhou University of Chinese Medicine, Guangzhou 510006, China

[2] State Key Laboratory of Oncology in South China, Guangdong Provincial Clinical Research Center for Cancer, Sun Yat-Sen University Cancer Center, Guangzhou 510060, China

[3] Center of Ganstric Cancer, The First Affiliated Hospital, Sun Yat-Sen University, Guangzhou 510062, China

[4] Dongguan Institute of Guangzhou University of Chinese Medicine, Dongguan 523808, China

[5] Shenzhen Traditional Chinese Medicine Hospital, The Fourth Clinical Medical College of Guangzhou University of Chinese Medicine, Shenzhen 518033, China

[†] These authors contributed equally to this work.

[#] **Corresponding authors:**

Yongming Chen (chenyongm@sysucc.org.cn); Wei Wang (wangwei26960@126.com); Hailin Tang (tanghl@sysucc.org.cn)

Supplementary Figures

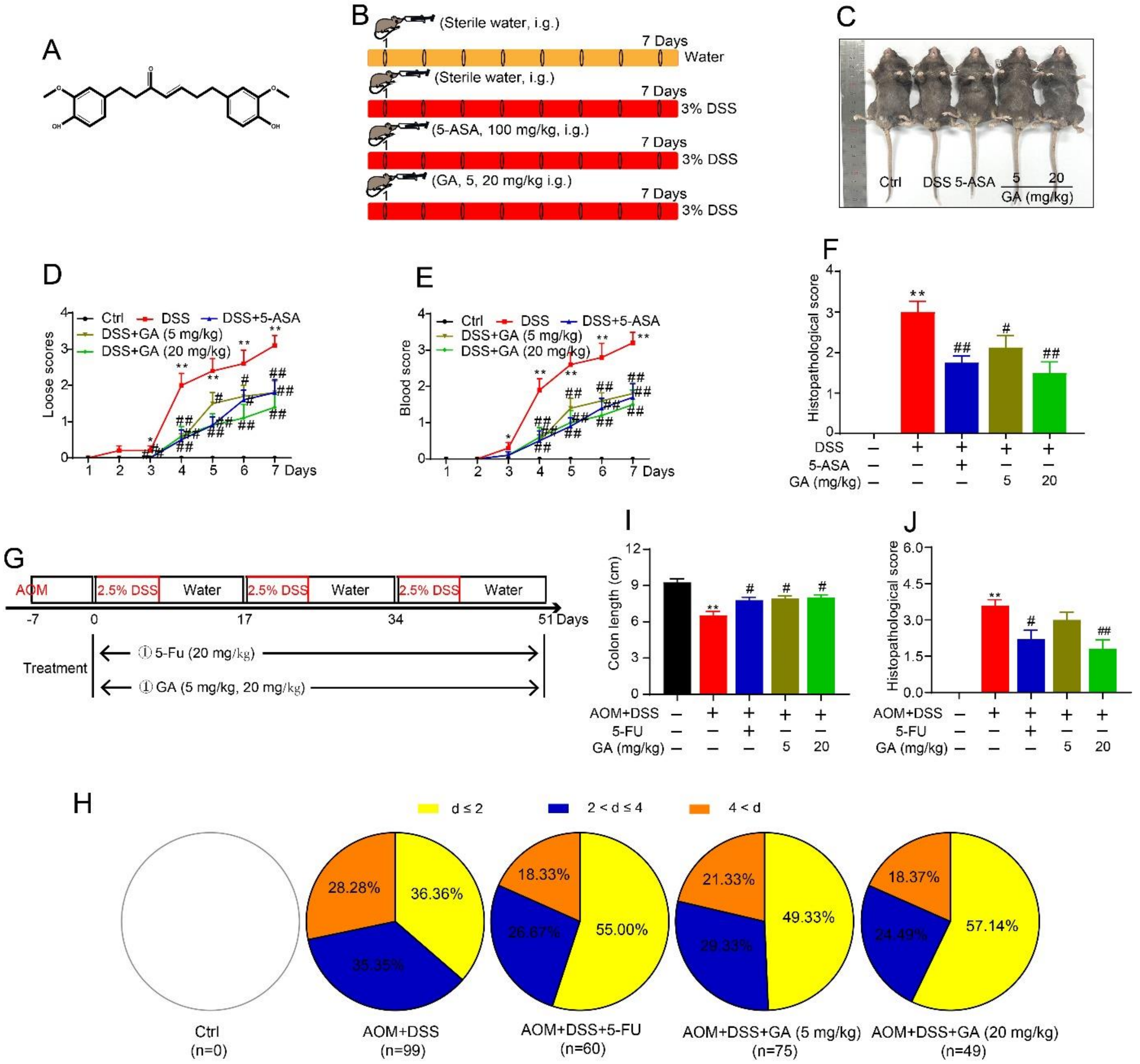

Figure S1. Effect of GA on DSS-induced colitis mice and AOM+DSS induced colonic tumorigenesis. (A) The chemical structure of Gingerenone A (GA). (B) The schedule of DSS-induced colitis mice, C57BL/6 mice were fed with water or 3% DSS for 7 days, and orally treated with 5-ASA (100 mg/kg) and GA (5 and 20 mg/kg) until the end of the experiment. (C) The representative images of gross appearance for colitis mice (*n*=10). (D) The loose stool score in DSS-induced colitis mouse model (*n*=10). (E) The blood stool score in DSS-induced colitis mouse model (*n*=10). (F) Colonic histopathological score in DSS-induced colitis mice. (G) The schedule of AOM/DSS induced colitis-associated cancer experiment, the mice were injected with the mutagen azoxymethane (AOM) for 7 days, after, mice were provided with 2.5% DSS-containing water for 7 days and recovered for 14 days, mice also were administered with GA or 5-Fu. (H) The number distribution of tumors with different diameters (*n*=5). (I) Colon length in AOM/DSS colitis-associated cancer model (*n*=5). (J)

Histopathological score of H&E staining in AOM/DSS colitis-associated cancer model (n=5). All data are expressed as mean ± S.E.M. $^{*}P < 0.05$ and $^{**}P < 0.01$ compared with the control group (Ctrl), $^{\#}P < 0.05$ and $^{\#\#}P < 0.01$ compared with the DSS group or AOM/DSS group.

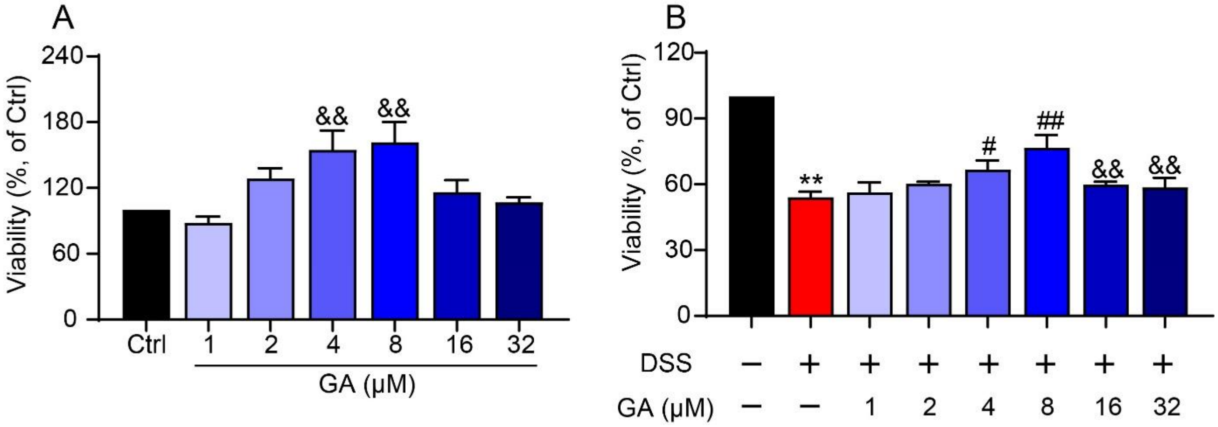

Figure S2. Effect of GA on viability in gut organoids. (A) The viability of organoids was evaluated in GA administration. (B) The viability of organoids was detected in GA administration and expose to DSS (0.01%) stimulation. All data are expressed as mean ± S.E.M (n=4). $^{*}P < 0.05$ and $^{**}P < 0.01$ (or $^{\&}P < 0.05$ and $^{\&\&}P < 0.01$) compared with the control group (Ctrl), $^{\#}P < 0.05$ and $^{\#\#}P < 0.01$ compared with the DSS group.

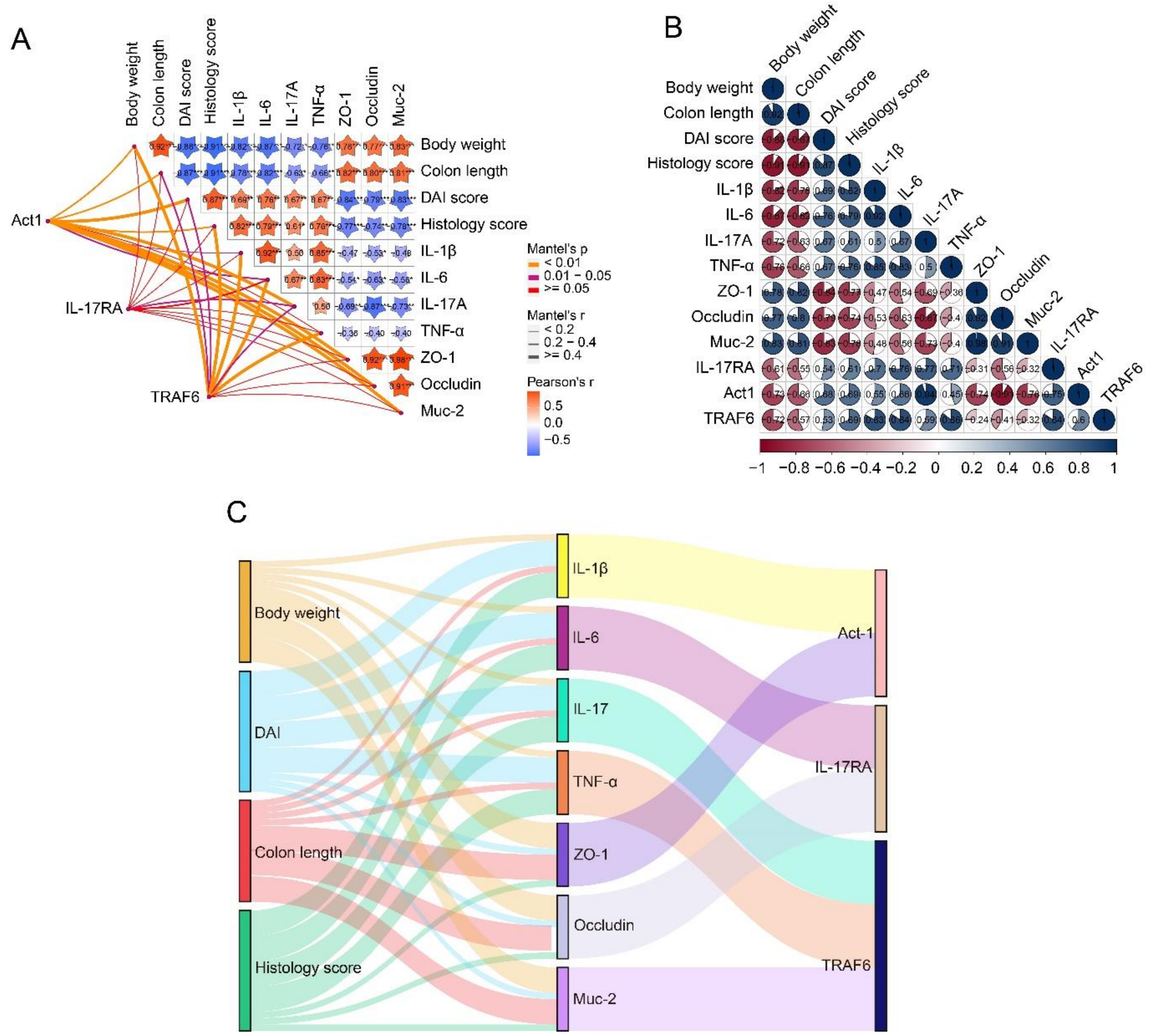

Figure S3. The correlation analysis between IL-17 signaling and colitis. (A) Mantel test analysis. (B) Corr_heatmap analysis. (C) Specific genes and imputed functional profiles (inflammation and intestinal barrier function) related to UC clinical symptoms. All data are expressed as mean ± S.E.M (*n*=3)

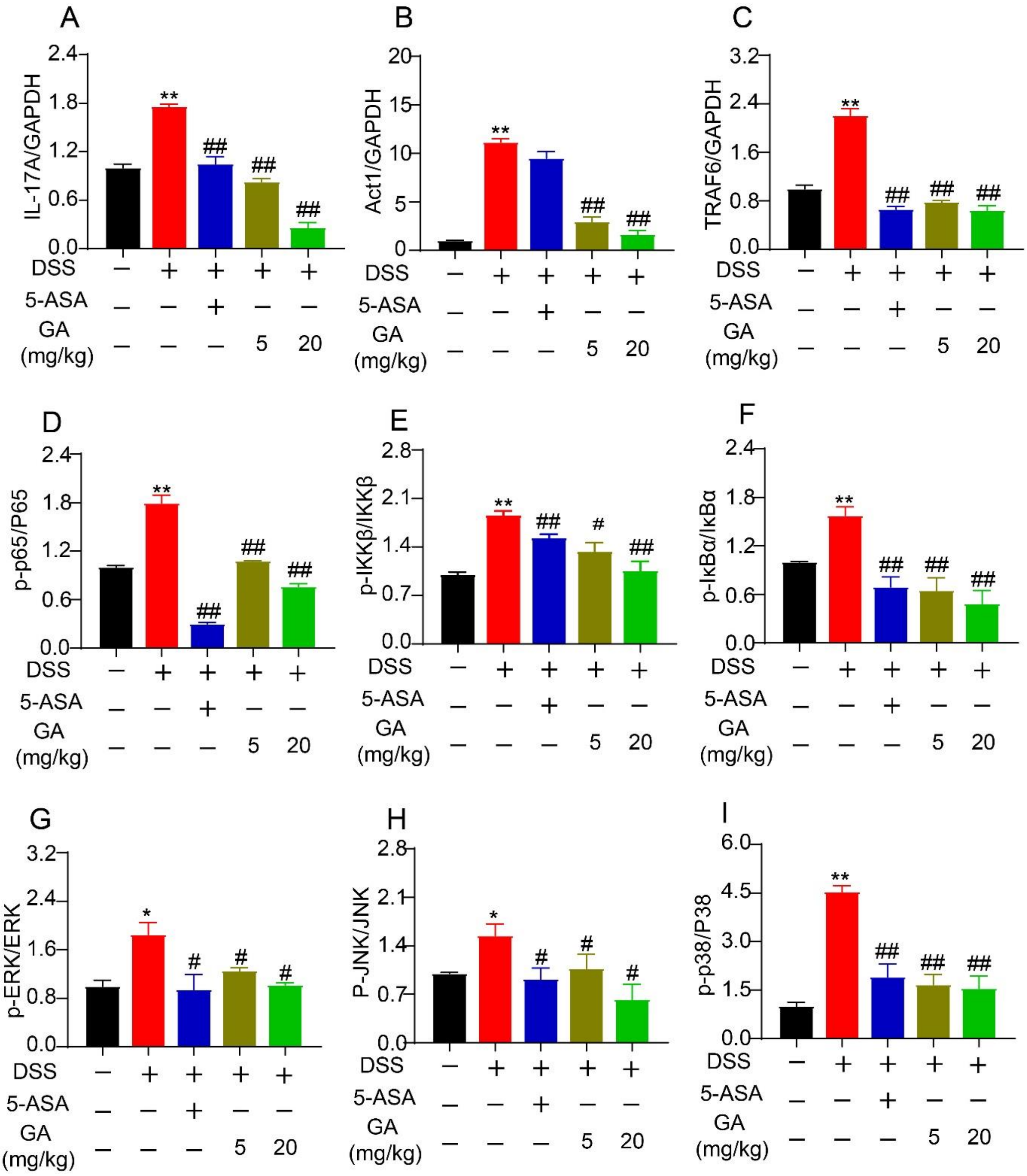

Figure S4. Effect of GA on inhibiting IL-17RA signaling and its downstream signaling in DSS-induced colitis mice. (A-I) The expressions of IL-17RA signaling (IL-17RA, Act1 and TRAF6) and its downstream signaling (p65, IκBα, p-IκBα, IKKβ, p-IKKβ, ERK, p-ERK, JNK, p-JNK, p38 and p-p38) proteins. All data are expressed as mean ± S.E.M (n=3). $^{*}P < 0.05$ and $^{**}P < 0.01$ compared with the control group (Ctrl), $^{\#}P < 0.05$ and $^{\#\#}P < 0.01$ compared with the DSS group.

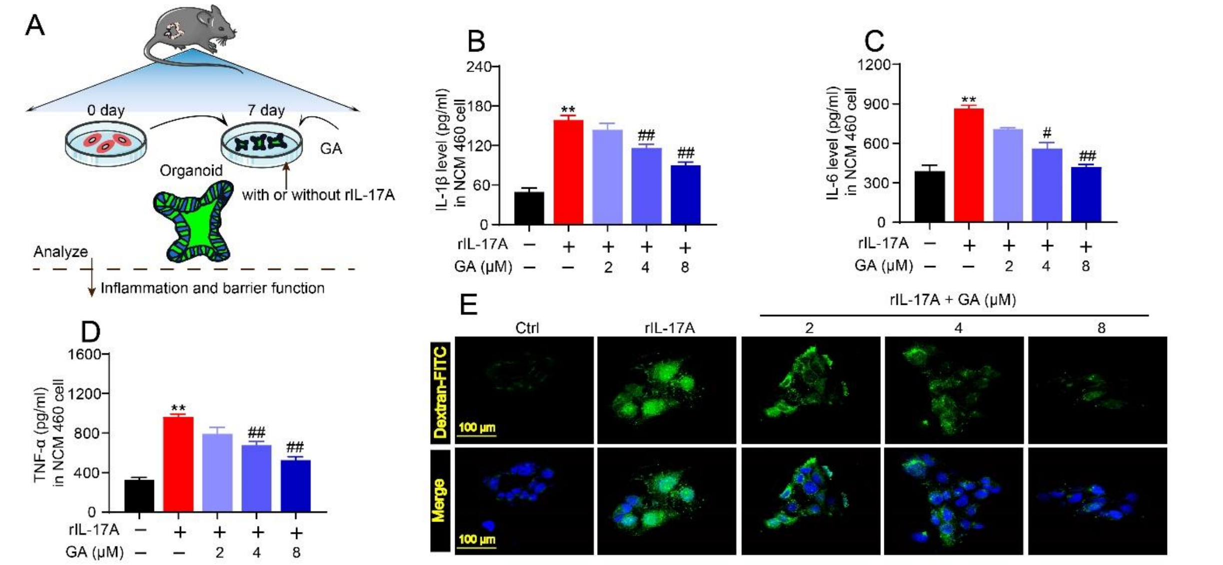

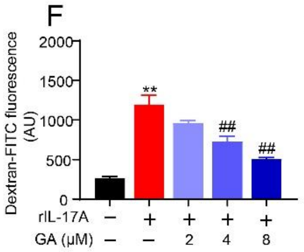

G

Group	Ctrl	rIL-17A	rIL-17A+GA (2 μM)	rIL-17A+GA (4 μM)	rIL-17A+GA (8 μM)
ZO-1 (%)	100.00 ± 5.08	21.90 ± 1.18**	30.06 ± 1.97	54.80 ± 2.64##	71.75 ± 1.93##
Occludin (%)	100.00 ± 3.53	26.25 ± 1.68**	30.14 ± 2.20	49.34 ± 3.31##	77.51 ± 2.72##
Muc-2 (%)	100.00 ± 3.42	14.72 ± 1.58**	18.16 ± 0.79	39.28 ± 1.88##	88.09 ± 0.98##

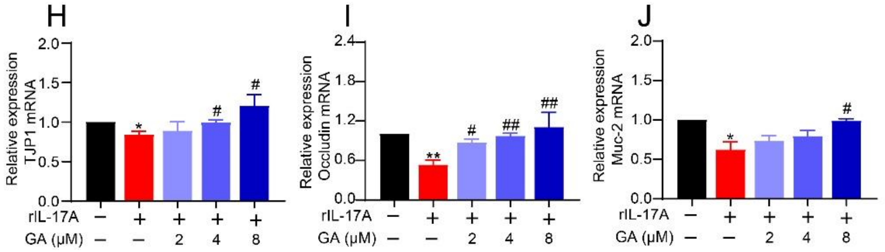

Figure S5. Effect of GA on inhibiting inflammation and maintaining epithelial integrity in rIL-17A-induced NCM460 cells. (A) Schematic representation of organoid generation and GA intervention. (B-D) The levels of IL-1β, IL-6 and TNF-α in supernatant were detected using ELISA kits in rIL-17A-induced NCM 460 cells. (E-F) Representative fluorescent images of Dextran-FITC and the fluorescence intensity in rIL-17A-induced NCM 460 cells. (G) The fluorescent intensities of ZO-1, Occludin and Muc-2 in the organoids. (H-J) The mRNA levels of TJP1, Occludin and Muc-2 in rIL-17A-induced NCM 460 cells. All data are expressed as mean ± S.E.M (n=4-6). $^{*}P < 0.05$ and $^{**}P < 0.01$ compared with the control group, $^{\#}P < 0.05$ and $^{\#\#}P < 0.01$ compared with the rIL-17A group.

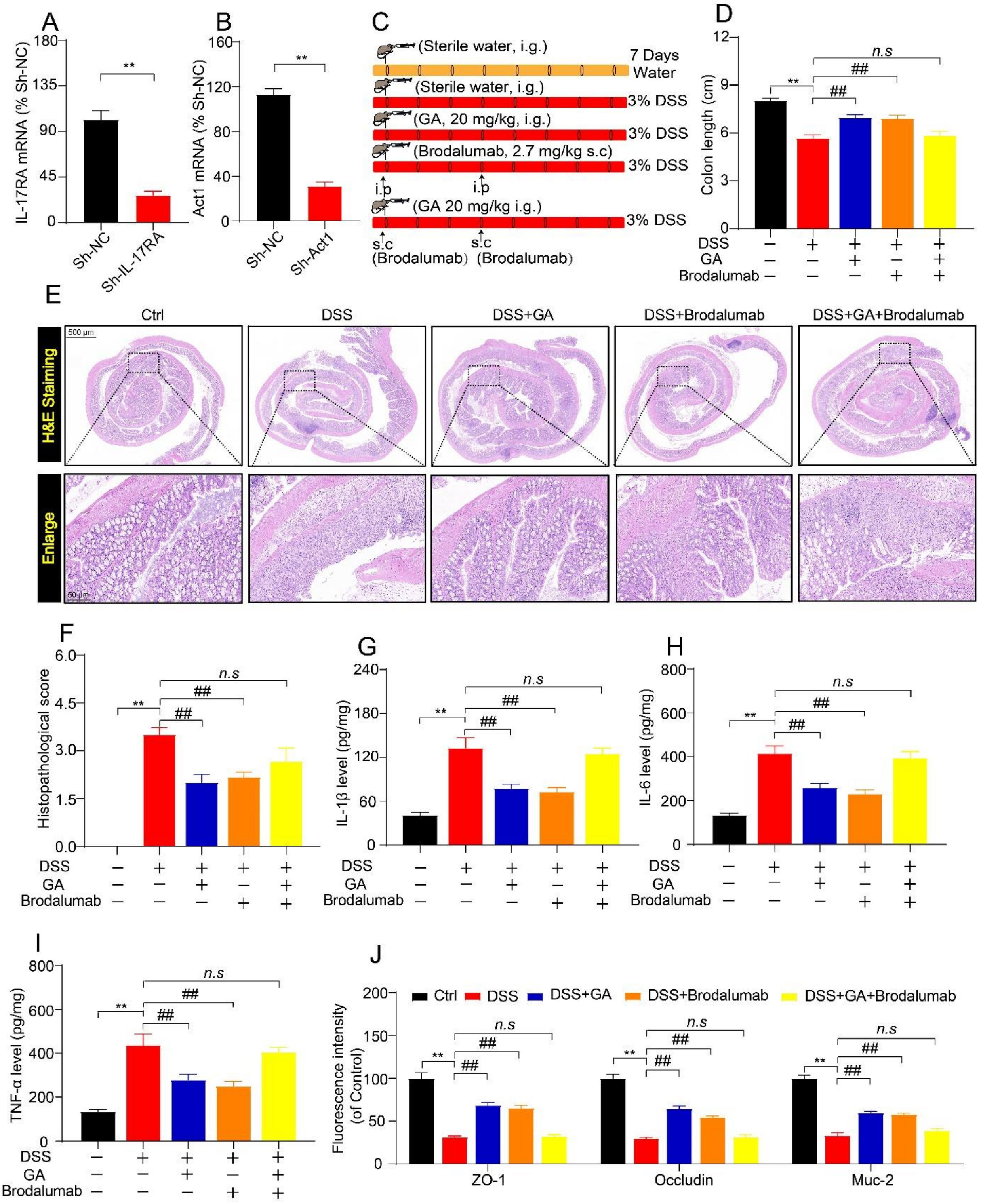

Figure S6. GA mitigated DSS-induced colitis mainly through suppressing IL-17RA signaling activation. (A) The knockdown efficacy of IL-17RA in organoids was detected using RT-PCR (*n*=4). (B) The knockdown efficacy of Act1 in organoids was detected using RT-PCR (*n*=4). (C) Mice were drunk with water or 3% DSS-containing water for 7 days, and treated with GA and brodalumab alone or in combination. (D) Colon length. (E-F) Representative images of colon tissues H&E staining and histopathological score. (G-I) The levels of pro-inflammatory cytokines (IL-1β, IL-6 and TNF-α) in colinic tissues. (J) The fluorescence intensity of ZO-1,

Occludin and Muc-2 in the colonic tissues of colitis mice. All data are expressed as mean ± S.E.M (*n*=6). $^{*}P < 0.05$ and $^{**}P < 0.01$ compared with the control group (or Sh-NC), $^{\#}P < 0.05$ and $^{\#\#}P < 0.01$ compared with the rIL-17A group. *n.s* indicates no significance.

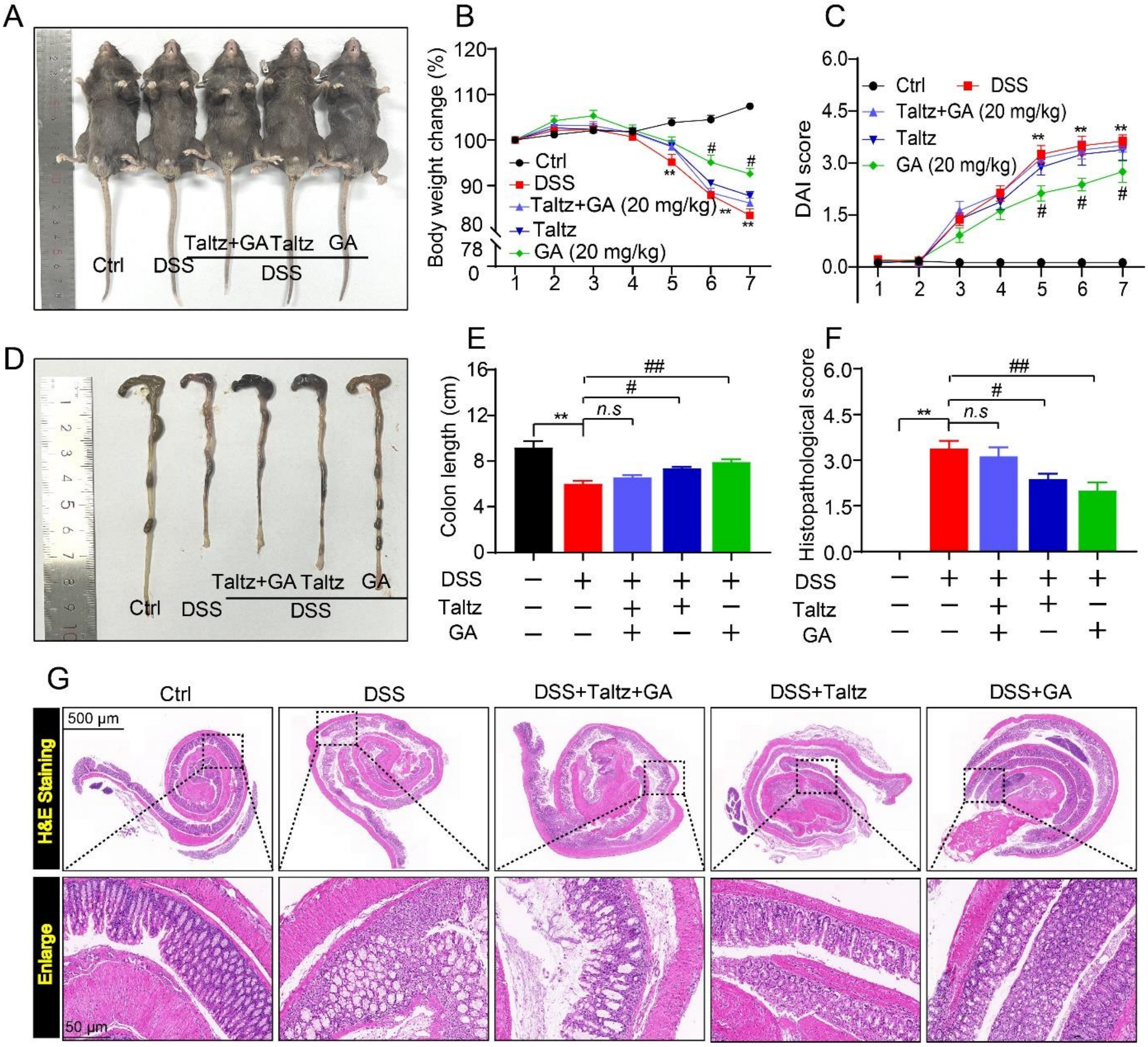

Figure S7. Ixekizumab (Taltz) counteracts the effect of GA on alleviating DSS-induced colitis. (A) The representative images of colitis mice somatotype. (B) Body weight change. (C) DAI score. (D) Representative images of colon for colitis mice. (E) Colon length. (F-G) Histopathological score and representative images of colonic tissues H&E staining. All data are expressed as mean ± S.E.M (*n*=6). $^{*}P < 0.05$ and $^{**}P < 0.01$ compared with the control group, $^{\#}P < 0.05$ and $^{\#\#}P < 0.01$ compared with the rIL-17A group. *n.s* indicates no significance.

Supplementary Table

Supplementary Table S1: Sequences of primers for quantitative real-time PCR.

Mouse genes		Sequence 5′–3′
IL-17RA	Forward	GACCAGGGCTACATCTCCA
	Revere	CAGGCTCCGTAGTTCCTCA
Act1	Forward	CTGGTGAACGCAAGAGGT
	Revere	GCAGGAAGTGGATGCTGT
CXCL10	Forward	AGCCGTGGTCACATCAG
	Revere	ATCCCAGCCACTTGAGC
CCL20	Forward	GTGTGCGCTGATCCAAA
	Revere	GGGCTGTGTCCAATTCC
CCL2	Forward	TTGTCACCAAGCTCAAGAGAGA
	Revere	GAGGTGGTTGTGGAAAAGGTAG
CCL7	Forward	CAGAAGTGGGTCGAGGAG
	Revere	AAGAACAGCGGTGAGGAA
Occludin	Forward	CTGCCTGCACGATGTCT
	Revere	GAGTGTTCAGCCCAGTCAA
Muc-2	Forward	TAAGCCACAACCCAGACC
	Revere	ACGGCAGAGCAGAAACA
Tjp1	Forward	TCAGCAGCAACAGAACCA
	Revere	GTAGCACCATCCGCCTT
